# Furin‐Mediated Cleavage of Zona Pellucida Proteins Is Essential for Oocyte Development

**DOI:** 10.1002/mco2.70542

**Published:** 2025-12-12

**Authors:** Tiechao Ruan, Xiang Wang, Xueguang Zhang, Yan Wang, Chuan Jiang, Sixian Wu, Yunchuan Tian, Xinyao Tang, Jun Ma, Shikun Zhao, Liangchai Zhuo, Mohan Liu, Siyu Dai, Zhenbo Wang, Wenming Xu, Ying Shen

**Affiliations:** ^1^ Department of Obstetrics/Gynecology Key Laboratory of Birth Defects and Related Disease of Women and Children of MOE West China Second University Hospital, Sichuan University Chengdu China; ^2^ Department of Pediatrics West China Second University Hospital, Sichuan University Chengdu China; ^3^ Reproduction Medical Centre West China Second University Hospital, Sichuan University Chengdu China; ^4^ State Key Laboratory of Stem Cell and Reproductive Biology Institute of Zoology Chinese Academy of Sciences Beijing China; ^5^ University of Chinese Academy of Sciences Beijing China; ^6^ NHC Key Laboratory of Chronobiology Sichuan University Chengdu China

**Keywords:** empty follicle syndrome, female infertility, furin cleavage, posttranslational modification, zona pellucida

## Abstract

Zona pellucida (ZP) proteins, essential for oocyte development, undergo posttranslational regulation through furin‐mediated cleavage. Nevertheless, our understanding of the functional significance of furin‐mediated cleavage of ZP proteins in female reproduction remains limited. Here, using mouse models with disrupted furin cleavage sites in ZP1, ZP2, and ZP3, we found that loss of the furin site in ZP2 caused female infertility associated with empty follicle syndrome (EFS), manifested by the failure to retrieve oocytes after ovarian hyperstimulation. In contrast, female mice carrying cleavage‐resistant variants at the furin sites of ZP1 and ZP3 exhibited defective ZP in a subset of oocytes, leading to reduced fecundity. Mechanistically, disruption of the furin cleavage site in ZP2 impaired the transmembrane transport of the non‐cleaved ZP2 protein and subsequently reduced the levels of SNARE proteins, ultimately triggering oocyte apoptosis through activation of the p53 and PI3K signaling pathways. Collectively, we uncovered the essential role of furin‐mediated cleavage of ZP proteins in female fertility and provided new mechanistic insights into the pathogenesis of EFS. These findings open new avenues for investigating the contribution of posttranslational modifications to female reproduction and for developing potential therapeutic strategies to treat female infertility.

## Graphical abstract

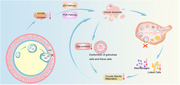

## 1 Introduction

Regular spermatogenesis and oogenesis are prerequisites for successful fertilization, which begins with the fusion of a healthy sperm cell and a mature oocyte. Prior to ovulation, each oocyte undergoes a series of growth and differentiation events to produce a mature oocyte capable of being fertilized. Briefly, the meiotic cell cycle of the oocyte is initiated in the embryonic ovary and becomes arrested at prophase I of meiosis [1]. Upon the onset of puberty, hormonal stimulation periodically triggers the resumption of meiosis in quiescent oocytes, leading to their progression from metaphase I to metaphase II [1, 2]. Remarkably, germinal vesicle (GV) breakdown (GVBD), extrusion of the first polar body, and asymmetric division are the most prominent features during this process [3, 4]. Importantly, any factor that interferes with these events may cause oocyte degeneration or developmental failure, ultimately resulting in female infertility [5].

The zona pellucida (ZP) is a thick extracellular matrix surrounding the oocyte and functions as the irreplaceable structural component of the oocyte [6]. The ZP plays indispensable roles in preimplantation development, fertilization, and oogenesis [7]. In humans, the ZP consists of four glycoproteins (ZP1–ZP4), whereas in mice, it is composed of three homologs (ZP1–ZP3) because *Zp4* is a pseudogene [8]. Female mice lacking *Zp1* develop a loosely organized matrix instead of a compact zona around their oocytes, resulting in subfertility due to early embryonic loss [9]. Knockout of *Zp2* or *Zp3* disrupts zona matrix assembly and folliculogenesis, leading to complete infertility in mice [10, 11]. In humans, pathogenic variants in *ZP* gene (*ZP1*–*ZP4)* have been linked to defective ZP assembly and compromised structural integrity, resulting in ovulated oocytes with a markedly thinned or even absent ZP [12]. Moreover, mutations in *ZP1*, *ZP2*, and *ZP3* genes have been associated with empty follicle syndrome (EFS), marked by the absence of oocytes within follicles, highlighting the essential roles of ZP proteins not only in fertilization but also in folliculogenesis [12, 13].

In addition to aberrant expression of ZP proteins, abnormal posttranslational modification (PTM) of ZP components has also been linked to female infertility. Both human and mouse ZP proteins include four main domains: a signal peptide, a zona domain, a consensus furin cleavage site (CFCS), and a transmembrane domain [8]. Furin site (RX^K^/_R_R) cleavage represents an essential PTM event mediated by furin, a transmembrane serine protease residing in the endomembrane system [14, 15]. However, its role in secretion and function of ZP proteins remains poorly understood. Limited and inconsistent studies have assessed the impact of furin cleavage on the extracellular transport of ZP3 [16, 17, 18, 19, 20]. Recently, a heterozygous variant within the CFCS of *ZP2* was shown to cause cytoplasmic retention of ZP2 and EFS phenotypes in both affected females and the corresponding knock‐in (KI) mouse model [21]. Moreover, oocyte‐specific deletion of furin results in oocyte dysplasia [22], emphasizing the crucial role of furin in oocyte development. Collectively, these findings suggest that furin‐mediated cleavage is indispensable for proper ZP assembly and female fertility, yet direct mechanistic evidence elucidating how furin‐dependent processing regulates ZP formation and oogenesis remains limited.

Here, we used CRISPR‐Cas9 to omit the furin cleavage sites of *Zp1*, *Zp2*, and *Zp3* in mouse models to explore the potential function of furin cleavage‐mediated PTM of ZP proteins in female reproduction. Notably, mouse models with impaired furin cleavage of ZP2 displayed infertility related to EFS phenotypes. Moreover, mice harboring mutations in the furin cleavage site of *Zp1* and *Zp3* produced partial oocytes with absent or defective ZPs and exhibited subfertility. Our study showed that furin cleavage of ZP proteins is crucial for oocyte development and loss of furin cleavage modification in ZP2 is especially responsible for EFS.

## 2 Results

### 2.1 Absence of Furin Cleavage in ZP2 Causes EFS Phenotypes and Female Infertility in Mice

**FIGURE 1:**
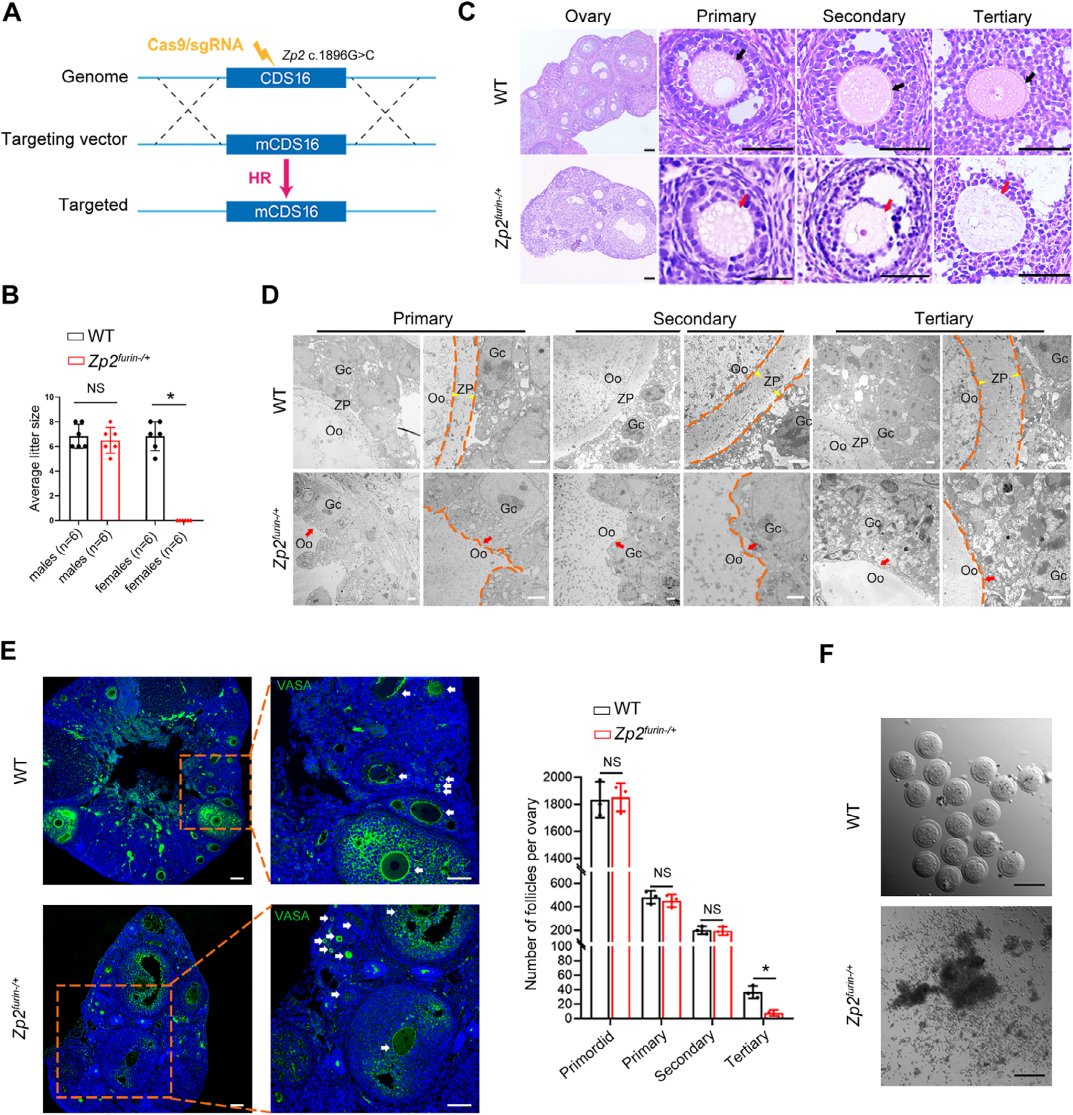
Absence of ZP2 furin site causes EFS phenotypes in mice. (A) Diagram illustrating the generation of *Zp2^furin−/+^* (*Zp2* c.1896G>C) mice. (B) The *Zp2^furin−/+^* females (*n* = 6) exhibited infertility phenotype, and *Zp2^furin−/+^* males (*n* = 6) were fertile. Student's *t*‐test; NS, not significant; *p < 0.05; error bars, s.e.m. (C) H&E staining of ovarian sections from WT (*n* = 3) and *Zp2^furin−/+^* (*n* = 3) females. Black arrows mark the follicles with an intact ZP. Red arrows indicate the abnormal ZP. Scale bars, 150 µm. (D) TEM showed the deficient ZP in oocytes from ovaries of *Zp2^furin−/+^* mice (*n* = 3) compared with WT mice (*n* = 3). White arrowheads indicate the normal ZP. Red arrows indicate the absent ZP. Double dashed lines outline ZP boundaries. Single dashed line delineates the interface between oocyte and granulosa cells. Scale bars, 2 µm. (E) VASA staining of ovaries showed ovarian follicles in *Zp2^furin−/+^* mice (*n* = 3) as well as WT mice (*n* = 3). White arrows denote follicles at different developmental stages. Green, VASA; blue, DAPI; scale bars, 150 µm. Quantification of follicles across developmental stages in WT and KI mice. Analysis of each sample was performed in triplicate. Student's *t*‐test; NS, not significant; *p < 0.05; error bars, s.e.m. (F) No superovulated oocytes were obtained from the *Zp2^furin−/+^* mice (*n* = 3). Scale bars, 80 µm. Gc, granulosa cells; Oo, oocyte; ZP, zona pellucida.

We initially introduced c. 1896G>C mutation into *Zp2* to generate heterozygous KI mice with a disrupted ZP2 furin site (*Zp2^furin−/+^*) (Figure 1A). The successful establishment of heterozygous *Zp2^furin−/+^* mice was confirmed by Sanger sequencing (Figure S1A). No apparent differences in appearance were observed between *Zp2^furin−/+^* mice and WT mice. However, after mating, *Zp2^furin−/+^* female mice produced no progeny, whereas *Zp2^furin−/+^* male mice exhibited normal fertility (Figure 1B). Ovaries from *Zp2^furin−/+^* females were substantially smaller than those from WT controls (Figure S1B).

Hematoxylin–eosin (H&E) staining showed that the ZP was barely detectable in oocytes from *Zp2^furin−/+^* female mice (Figure 1C), which was further confirmed by transmission electron microscopy (TEM) (Figure 1D and Figure S1C). VASA staining demonstrated a significant decrease in tertiary follicles in *Zp2^furin−/+^* female mice (Figure 1E). Strikingly, no oocytes except some granulosa cells were retrieved from the *Zp2^furin−/+^* female mice after ovarian hyperstimulation (Figure 1F). These findings collectively resembled the phenotype of EFS.

**FIGURE 2:**
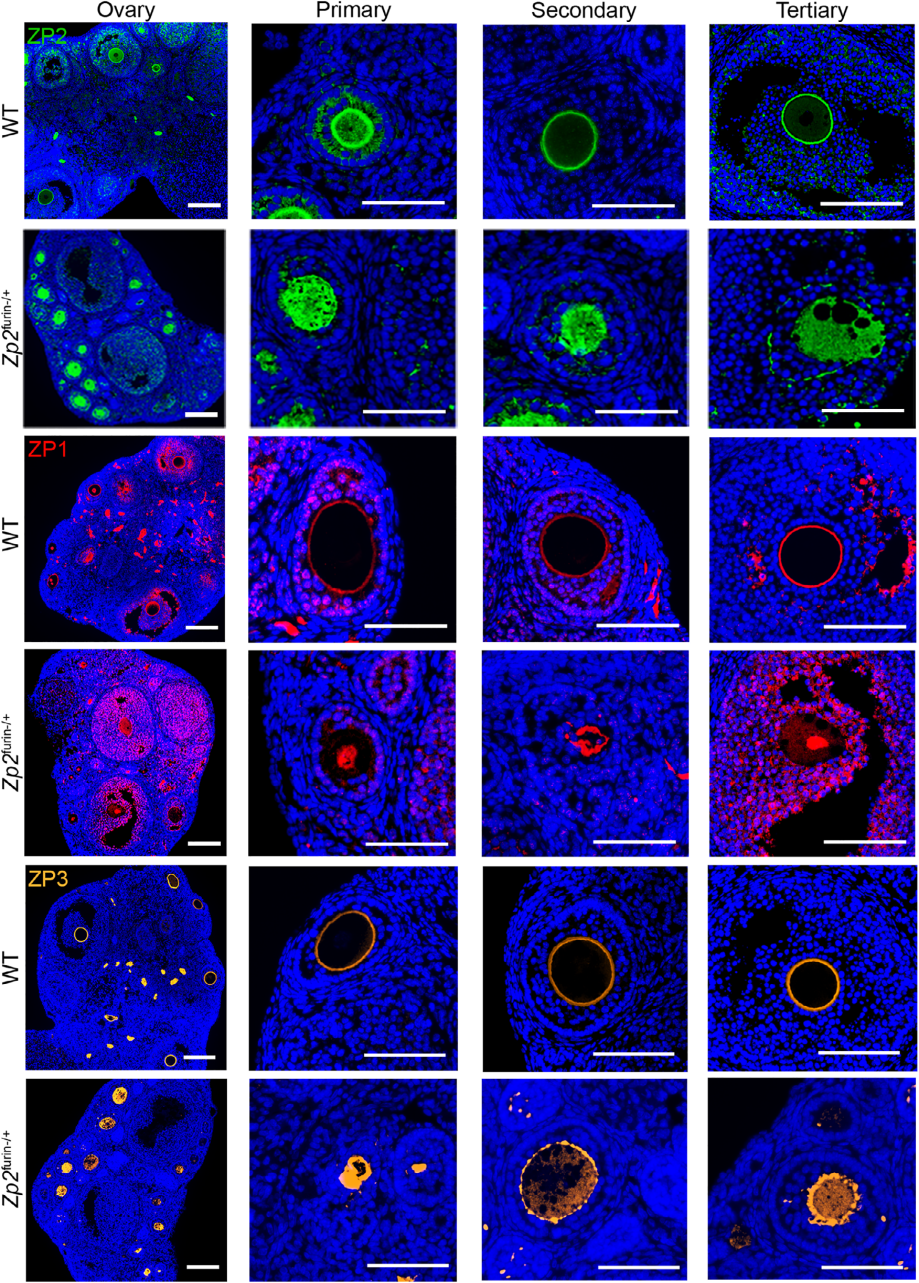
The abnormal localization of ZP proteins in oocytes of *Zp2* *^furin−/+^* **mice**. Immunofluorescence staining of ZP1, ZP2, and ZP3 in ovaries of *Zp2^furin−/+^* mice (*n* = 3). Blue, DAPI; red, ZP1; green, ZP2; orange, ZP3; scale bars, 300 µm. ZP, zona pellucida.

Immunofluorescence analysis of ovarian sections further indicated cytoplasmic accumulation of ZP2 in *Zp2^furin−/+^* oocytes (Figure 2 and Figure S1D). Notably, ZP1 and ZP3 proteins were likewise mislocalized within oocytes from *Zp2^furin−/+^* females (Figure 2 and Figure S1E,F). Together, these findings suggested that disruption of furin‐mediated cleavage of ZP2 impairs ZP formation and perturbs the proper localization of ZP1 and ZP3, ultimately leading to female infertility consistent with EFS.

### 2.2 Abolishment of Furin Cleavage in ZP1 and ZP3 Interferes With Normal ZP Development

**FIGURE 3:**
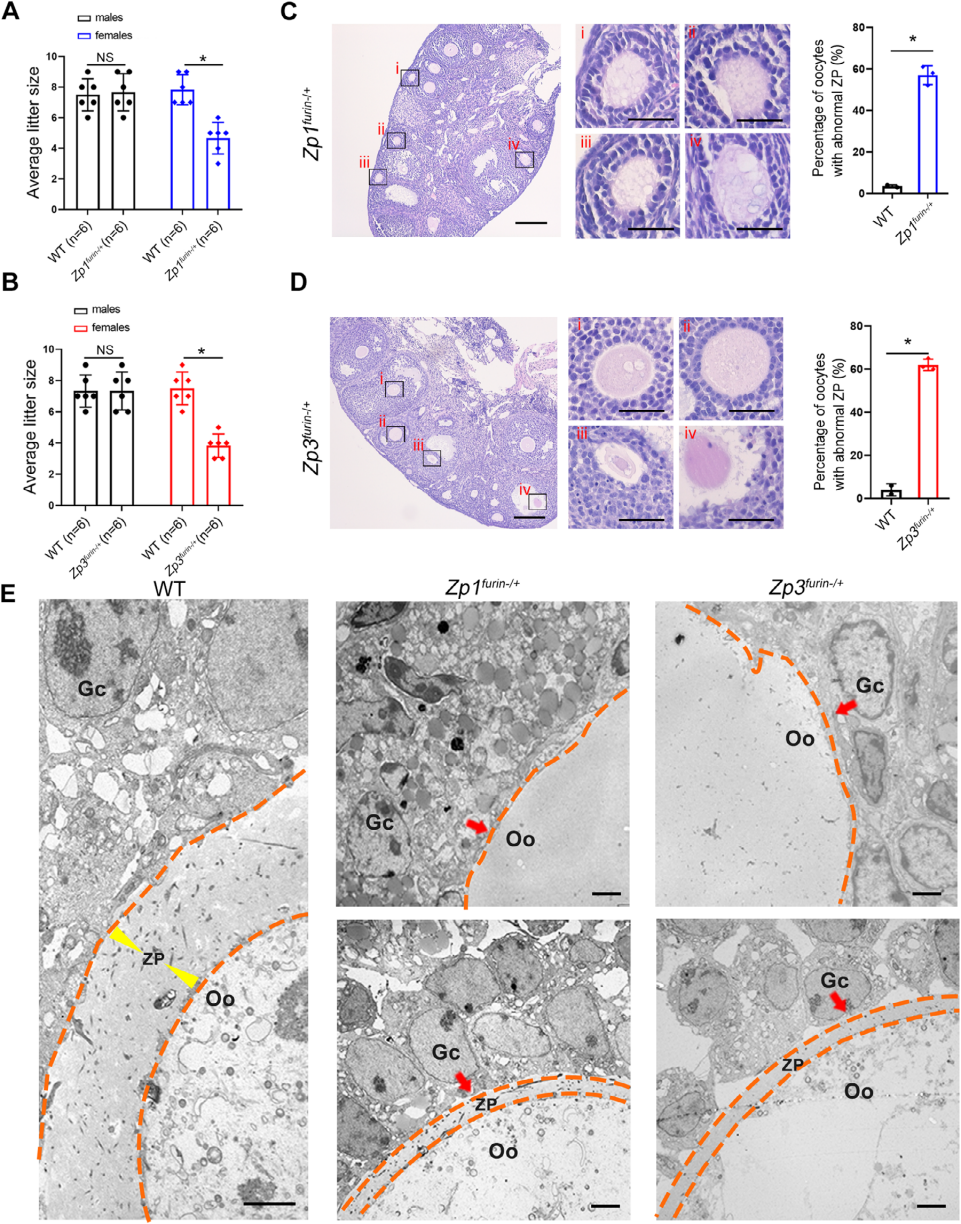
Lacking furin‐mediated cleavage in ZP1 and ZP3 leading to defects in mouse oocytes. (A and B) *Zp1^furin−/+^* female (*n* = 6) (A) and *Zp3^furin−/+^* female mice (*n* = 6) (B) exhibited subfertility phenotypes. Student's *t*‐test; NS, not significant; *p < 0.05; error bars, s.e.m. (C and D) H&E staining found some oocytes of *Zp1^furin−/+^* mice (C) or *Zp3^furin−/+^* mice (D) showing the missing ZP. Statistical analysis of oocytes with defective ZPs in *Zp1^furin−/+^* mice (*n* = 3) and *Zp3^furin−/+^* mice (*n* = 3). Student's *t*‐test; *p < 0.05; error bars, s.e.m. Scale bar, 50 µm. (E) TEM analysis detected loss or marked thinning of the ZP in partial oocytes from *Zp1^furin−/+^* mice (*n* = 3) or *Zp3^furin−/+^* mice (*n* = 3). Red arrows indicate the absent ZP. Double dashed lines outline ZP boundaries. Single dashed line delineates the interface between oocyte and granulosa cells. Scale bars, 2 µm. Gc, granulosa cells; Oo, oocyte; ZP, zona pellucida.

We further generated mouse models lacking the furin cleavage site of ZP1 (*Zp1^furin−/+^*) and ZP3 (*Zp3^furin−/+^*), as *Zp4* is absent in mice (Figure S2A,B). Genotypes were validated by Sanger sequencing (Figure S2C,D). Although ovarian sizes in *Zp1^furin−/+^* and *Zp3^furin−/+^* female mice were comparable to those in WT mice (Figure S2E,F), a significant reduction in average litter size in both strains indicated subfertility (Figure 3A,B).

H&E staining of ovarian sections revealed that a subset of oocytes lacked a ZP in *Zp1^furin−/+^* and *Zp3^furin−/+^* female mice (Figure 3C,D). Nevertheless, follicle numbers across different developmental stages were similar to those in WT mice (Figure S2G,H). Consistently, defective ZP structures were confirmed by TEM in both *Zp1^furin−/+^* and *Zp3^furin−/+^* mice (Figure 3E and Figure S2I).

**FIGURE 4:**
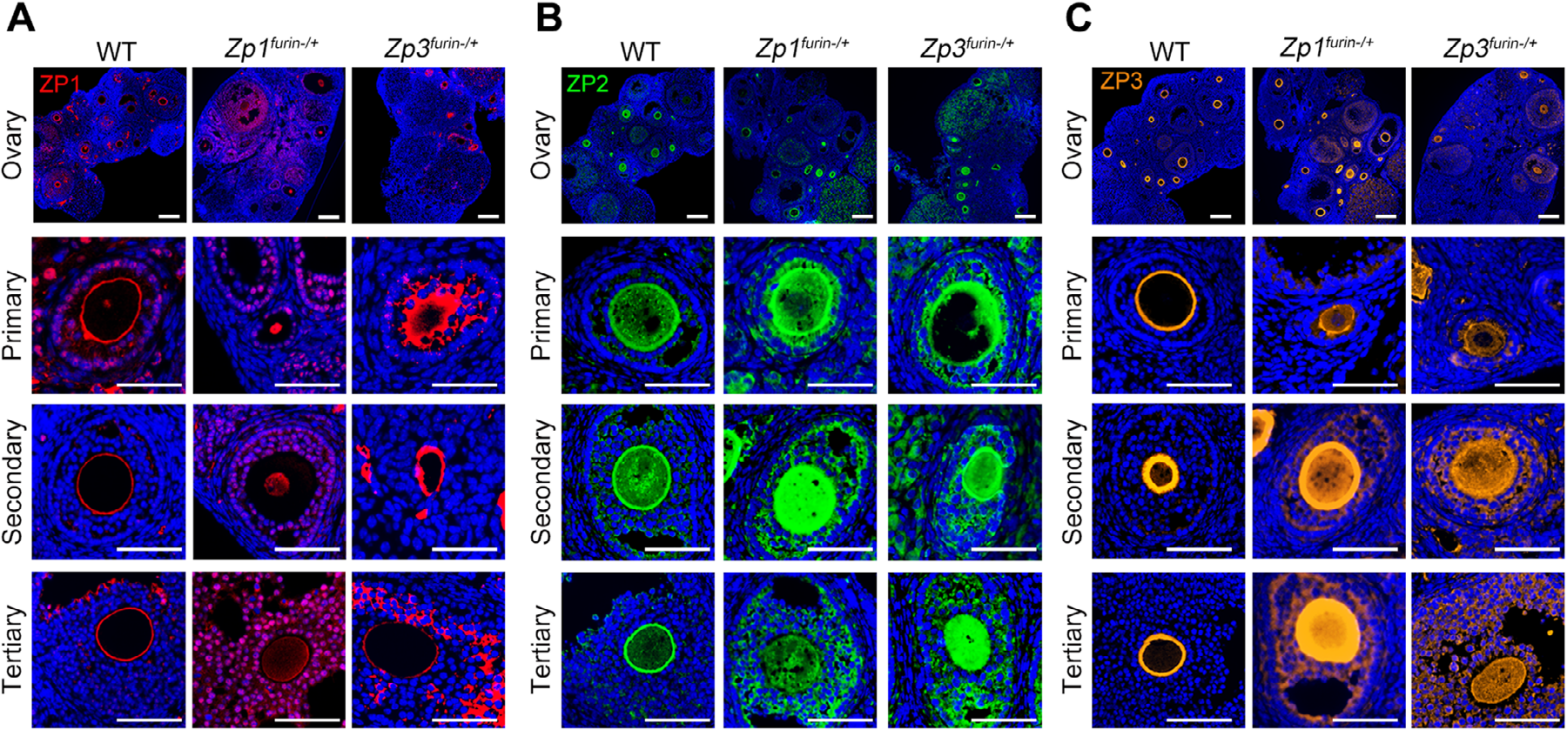
The anomalous expression of ZP proteins in heterozygous *Zp1* *^furin−/+^* **mice and *Zp3*** *^furin−/+^* **mice**. (A–C) Immunofluorescent staining of ZP1 (A), ZP2 (B), and ZP3 (C) represented irregular expression in some oocytes from *Zp1^furin−/+^* (*n* = 3) and *Zp3^furin−/+^* mice (*n* = 3). Blue, DAPI; red, ZP1; green, ZP2; orange, ZP3; scale bar, 300 µm. ZP, zona pellucida.

Furthermore, ZP1 accumulation within oocyte cytoplasm was observed in *Zp1^furin−/+^* mice (Figure 4A), whereas *Zp3^furin−/+^* female displayed ZP3 retention within the oocytes (Figure 4C). Remarkably, aberrant immunofluorescence patterns of ZP2 and ZP3 were found in *Zp1^furin−/+^* mice (Figure 4B,C), and *Zp3^furin−/+^* mice exhibited cytoplasmic retention of ZP1 and ZP2 (Figure 4A,B).

In contrast to the complete oocyte abnormality observed in mice with impaired ZP2 furin cleavage, *Zp1^furin−/+^* and *Zp3^furin−/+^* mice still presented several normally developed oocytes, indicating that additional enzymes may contribute to cleavage of the mutated furin cleavage site of ZP1 and ZP3. Thus, inhibition of furin‐mediated cleavage of ZP proteins contributes to female infertility, with loss of ZP2 furin cleavage being specifically responsible for the occurrence of EFS.

### 2.3 ZP2 Without Furin Cleavage is Defective in Extracellular Secretion

**FIGURE 5:**
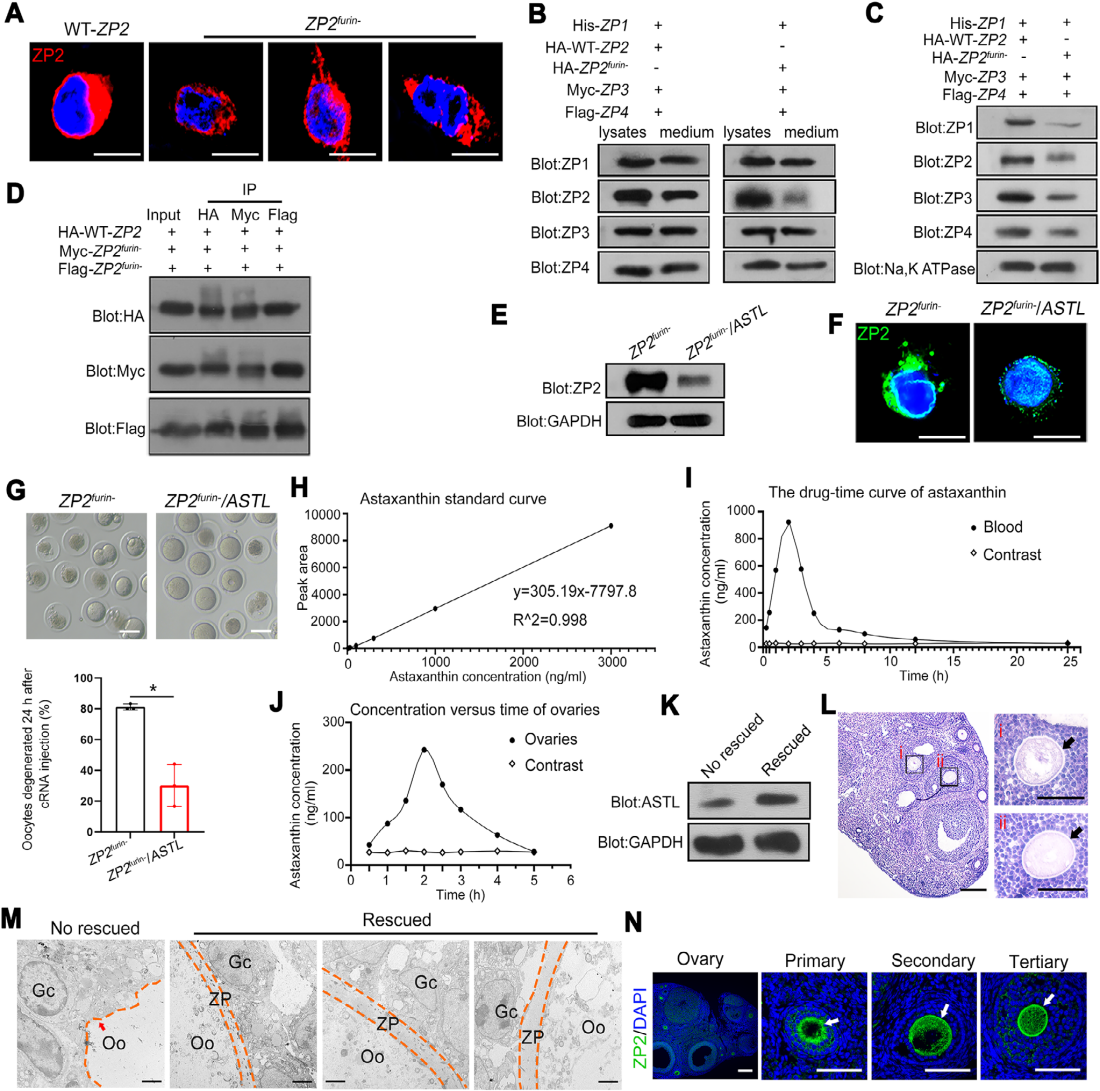
The impaired extracellular secretion of ZP2 protein with disrupted furin site. (A) Immunofluorescence staining of *ZP2* *^furin−^* and WT‐*ZP2* overexpressed in CHO‐K1 cells. Data shown are representative of three independent experiments. Blue, DAPI; red, ZP2; scale bars, 10 µm. (B) Western blot analysis of ZP1, ZP2, ZP3, and ZP4 expressions in the medium and cell lysates. ZP1, ZP3, and ZP4 were each co‐expressed with either WT‐*ZP2* or *ZP2^furin−^* in CHO‐K1 cells, and proteins in the medium and cell lysates were then analyzed. The obvious signals of ZP1, ZP3, and ZP4 were observed in both media and cell lysates from the cells co‐transfected with WT‐*ZP2* or *ZP2^furin−^* plasmid, whereas extremely little ZP2 protein was detected in the media. Data shown are representative of three independent experiments. (C) Western blot analysis of membrane‐localized ZP1, ZP2, ZP3, and ZP4. *ZP1*, *ZP3*, and *ZP4* plasmids were co‐transfected with WT‐*ZP2* or *ZP2^furin−^* plasmids into CHO‐K1 cells, respectively. Plasma membrane protein was isolated from the transfected cells. The decreased ZP1, ZP2, ZP3, and ZP4 were detected inmembrane extracts from cells co‐transfected with *ZP2 ^furin−^* plasmid compared with WT‐*ZP2* control. Data shown are representative of three independent experiments. (D) Co‐IP assay of the interaction of WT‐*ZP2* and ZP2*^furin−^* WT‐*ZP2* plasmid (HA‐WT‐*ZP2*) and *ZP2^furin−^* plasmids (Myc‐*ZP2 ^furin−^* and Flag‐*ZP2^furin−^*) were co‐transfected into the CHO‐K1 cells. Data shown are representative of three independent experiments. (E) Western blot analysis of mutant ZP2 levels in cells transfected with or without *ASTL* plasmid. Data shown are representative of three independent experiments. (F) Immunofluorescence staining of mutant ZP2 in cells transfected with or without *ASTL* plasmid. Results are representative of three independent experiments. Green, ZP2; blue, DAPI; scale bars, 10 µm. Results are representative of three independent experiments. (G) Light microscopy of mouse GV oocytes at 24 h after the injection with *ZP2^furin−^* cRNAs or *ZP2^furin−^/ASTL* cRNAs. Statistical evaluation of oocyte degeneration following microinjection with *ZP2^furin−^* cRNAs or *ZP2^furin−^/ASTL* cRNAs. Student's *t*‐test; *p < 0.05; error bars, s.e.m. Scale bars, 100 µm. Data shown are representative of three independent experiments. (H) The standard curve of astaxanthin. (I) Plasma astaxanthin concentration‐time profiles by UPLC. (J) Ovaries astaxanthin concentration‐time profiles by UPLC. (K) Western blot analysis showed the increased expression of ASTL in rescued mice. (L) H&E staining of ovaries from *Zp^furin−/+^* mice (*n* = 3) after the treatment with astaxanthin. i and ii showed the oocytes with ZP. The ZP was indicated by black arrows; scale bars, 300 µm. (M) TEM analysis showed ZP in the oocytes from rescued *Zp2^furin−/+^* mice (*n* = 3) compared to the no rescued mice (*n* = 3). Red arrows indicate the absent ZP. Double dashed lines outline ZP boundaries. Single dashed line delineates the interface between oocyte and granulosa cells. Scale bars, 2 µm. (N) Immunofluorescence analysis of ZP2 in the ovaries of *Zp2^furin−/+^* mice (*n* = 3) after the treatment with astaxanthin. The location of ZP2 on ZP was indicated by white arrows; blue, DAPI; green, ZP2; scale bars, 300 µm. Gc, granulosa cells; Oo, oocyte; ZP, zona pellucida.

Currently, the pathological mechanism of EFS remains poorly defined. In our study, we demonstrate that disruption of furin‐mediated cleavage of ZP2 causes EFS in mice. To investigate the mechanism linking EFS and abnormal furin‐mediated cleavage of ZP2, we transfected CHO‐K1 cells with WT‐*ZP2* and *ZP2^furin−^* (c.1917G>C, p.R639S) plasmids. Immunofluorescence staining revealed that expression of *ZP2^furin−^* resulted in cytoplasmic aggregation of ZP2 in CHO‐K1 cells (Figure 5A), resembling the phenotype observed in *Zp2^furin−/+^* female mice. We further found that this accumulation resulted from a defective secretion process (Figure 5B), rather than defective intracellular trafficking (Figure S3A).

The secretion defect of mutant ZP2 did not alter the extracellular secretion of other ZP proteins (Figure 5B). However, it resulted in a reduction of ZP1, ZP3, and ZP4 on the plasma membrane (Figure 5C). Given that mutant proteins could interfere with WT protein function through interactions in some autosomal dominant hereditary diseases [23], we hypothesized that interactions between WT‐ZP2 and mutant ZP2 proteins impaired the transmembrane transport of WT‐ZP2 proteins reducing ZP2 secretion (Figure 5D and Figure S3B). Additionally, transfection of mutant plasmids of *ZP1^furin−^* (c.1664G>A) and *ZP3^furin−^* (c.903G>C) into CHO‐K1 cells resulted in moderate cytoplasmic accumulation of ZP1 and ZP3, respectively (Figure S3C,D). Together, these results suggested that abolishment of furin cleavage led to ZP2 protein retention in the cytoplasm due to defective extracellular secretion, thereby causing insufficient assembly of other ZP proteins on the plasma membrane.

### 2.4 The Ovastacin Effectively Cleaved the Accumulated ZP2 Protein

In this study, we indicated that loss of the furin cleavage site in ZP2 results in the cytoplasmic accumulation of nascent ZP2, leading to EFS in mice. Given that ZP2 also contains an alternative cleavage site for the astacin‐family metalloendoproteases, we hypothesized that oocyte dysplasia caused by impaired furin‐mediated cleavage might be alleviated through cleavage of the mutant ZP2 by other enzymes.

Ovastacin (also known as SAS1B), an astacin‐family metalloendoprotease encoded by *ASTL*, is released from cortical granules and cleaves ZP2 to block polyspermy [24]. The astacin cleavage site lies upstream of the furin site. Cleavage of the astacin cleavage site in ZP2 can remove the C‐terminus of ZP2, potentially facilitating degradation of the cleaved fragments or promoting export from the cytoplasm.

To test this hypothesis, we transfected CHO‐K1 cells with mutant *ZP2* plasmids alone or in combination with an *ASTL* expression plasmid. Western blotting showed a distinct decrease in the ZP2 level when ASTL and mutant ZP2 were co‐expressed (Figure 5E). Immunofluorescence staining further showed reduced cytoplasmic ZP2 staining and discernible ZP2 at the plasma membrane in the co‐transfected cells (Figure 5F).

We next wondered whether ovastacin could rescue mutant ZP2 dysfunction in oocytes. Mouse GV oocytes were microinjected with cRNAs (*ZP2^furin−^* alone or *ASTL* plus *ZP2^furin−^*). Oocytes receiving *ZP2^furin−^* cRNAs alone were significantly degenerated, whereas co‐injected with *ASTL* and *ZP2^furin−^* cRNAs alleviated the degeneration (Figure 5G).

Given these in vitro results, we evaluated an in vivo strategy. Astaxanthin is a natural astacin most commonly found in red carotenoids of marine and aquatic animals. Astaxanthin has been reported to upregulate ASTL expression in certain contexts [25, 26]. We therefore administered astaxanthin by continuous daily oral gavage to *Zp2^furin−/+^* females (16 mg/mL) for 3 months starting at 3 weeks of age. The pharmacokinetic data of astaxanthin indicated the presence of astaxanthin in mouse blood by ultraperformance liquid chromatography (UPLC) (Figure 5H,I and Table S1). We also confirmed that exogenous astaxanthin could reach the ovarian tissues using UPLC analysis (Figure 5J). As predicted, the ASTL protein levels increased in ovaries from rescued mice compared with no rescued mice (Figure 5K). Furthermore, astaxanthin ameliorated oocyte defects, restoring a discernible ZP in a subset of oocytes in rescued *Zp2^furin−/+^* mice (Figure 5L,M). In agreement with the in vitro results, mutant ZP2 proteins could be largely cleared from the oocyte cytoplasm in the rescued *Zp2^furin−/+^* mice (Figure 5N). Collectively, these data demonstrated that proteolytic removal of mutant ZP2 partially restores ZP2 secretion in cultured oocytes/cells and in *Zp2^furin−/+^* mice. Removing the excess cytoplasmic ZP2 provides a feasible mechanistic avenue to mitigate EFS caused by loss of ZP2 furin cleavage, warranting further investigation.

### 2.5 ZP2 With Disrupted Furin Site Alters Expression of Oogenesis‐Related Genes

**FIGURE 6:**
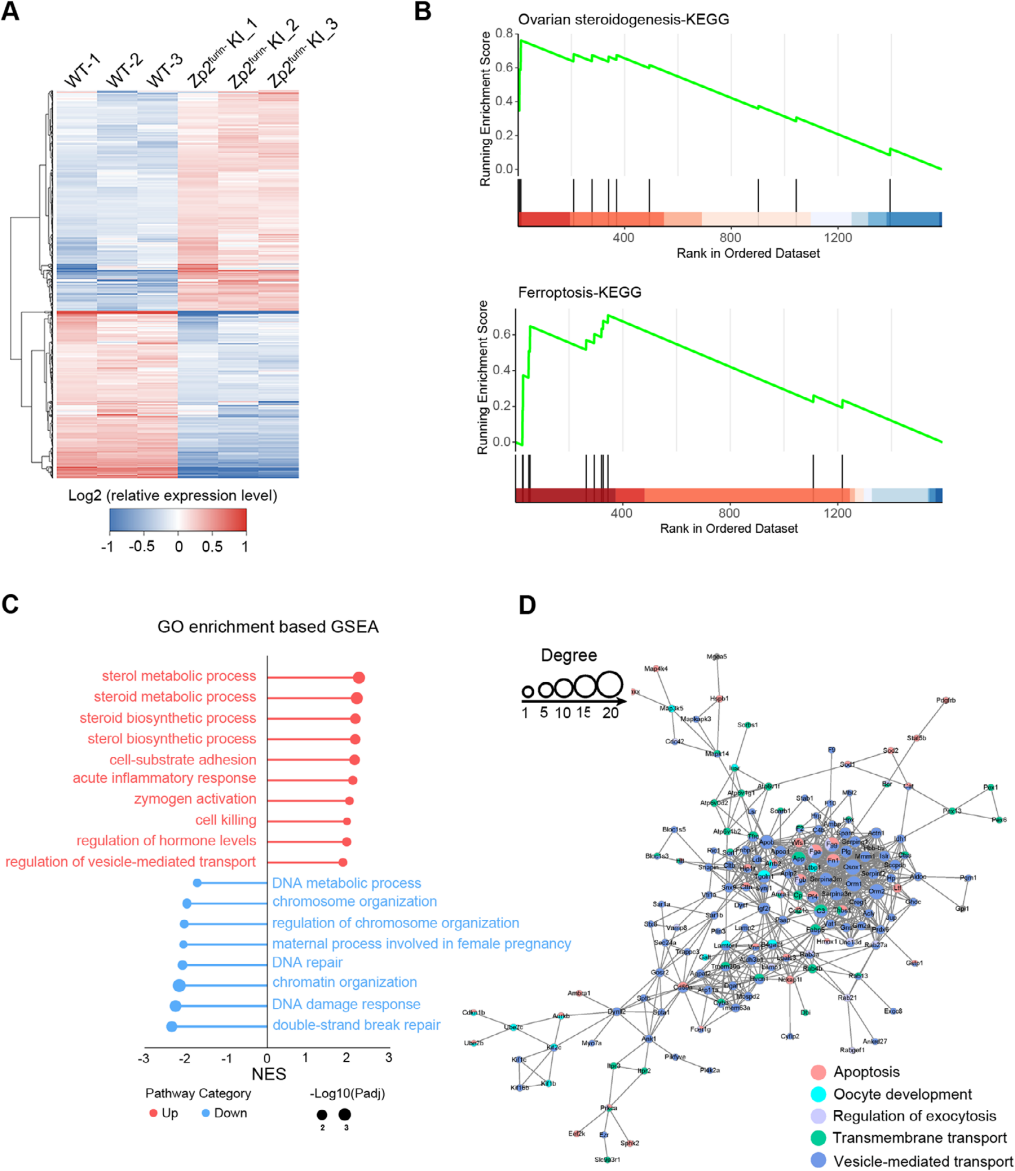
The differential expression genes related to oogenesis in *Zp2* *^furin^* ***^−^^/+^* and WT mice**. (A) The heat map from proteomic analysis of the ovaries from WT and *Zp2^furin−/+^* mice. (B) KEGG pathway‐based GSEA plots showing significant enrichment of proteins associated with ovarian steroidogenesis and ferroptosis in *Zp2^furin−^* ^/+^ mice compared with WT controls. (C) GO enrichment‐based GSEA analysis highlighting the upregulated processes (red) such as sterol metabolic process, zymogen activation, and cell killing, and the downregulated processes (blue) including chromatin organization and DNA repair. Circle size represents—log_10_(Padj). Padj: adjusted *p* value. (D) Interaction network analysis of differential proteins associated with oocyte development, oocyte apoptosis, and regulation of exocytosis, transmembrane transport, and vesicle‐mediated transport using the String database. Padj, adjusted *p* value; ZP, zona pellucida.

Cytoplasmic accumulation of non‐modified ZP2 in the oocyte directly triggers the onset of EFS. To investigate the underlying protein network alterations caused by the lack of furin cleavage in ZP2, we employed proteomics analysis of ovaries from *Zp2^furin−/+^* and WT mice. In total, 1908 proteins were quantified, of which 1081 were upregulated and 827 were downregulated in *Zp2^furin−/+^* mice (Figure 6A and Figure S4A). Importantly, KEGG pathway‐based GSEA analysis revealed significant alterations in proteins associated with ovarian steroidogenesis and the ferroptosis pathway in *Zp2^furin−/+^* mice (Figure 6B). Moreover, gene ontology (GO) enrichment‐based GSEA analysis demonstrated differentially expressed proteins relative to sterol metabolic process, zymogen activation, cell killing, and chromatin organization (Figure 6C). Notably, we constructed a protein–protein interaction network of the differentially expressed proteins involved in oocyte development, oocyte apoptosis, and the regulation of exocytosis, transmembrane transport, and vesicle‐mediated transport between *Zp2^furin−/+^* and wild‐type mice (Figure 6D). Western blotting and immunofluorescence staining were further used to confirm the aberrant expression of LHCGR, FDPS, KMT5B, GALT, RUNX2, MAP3K5, UBE2C, DAPK3, KIF1B, and BAX in the ovaries of *Zp2^furin−/+^* mice, all of which are essential for oogenesis (Figure S4B,C) [27, 28, 29, 30, 31, 32, 33, 34, 35, 36, 37].

### 2.6 Cell Pathologic Features of EFS Caused by Abolishment of Furin Cleavage in ZP2

**FIGURE 7:**
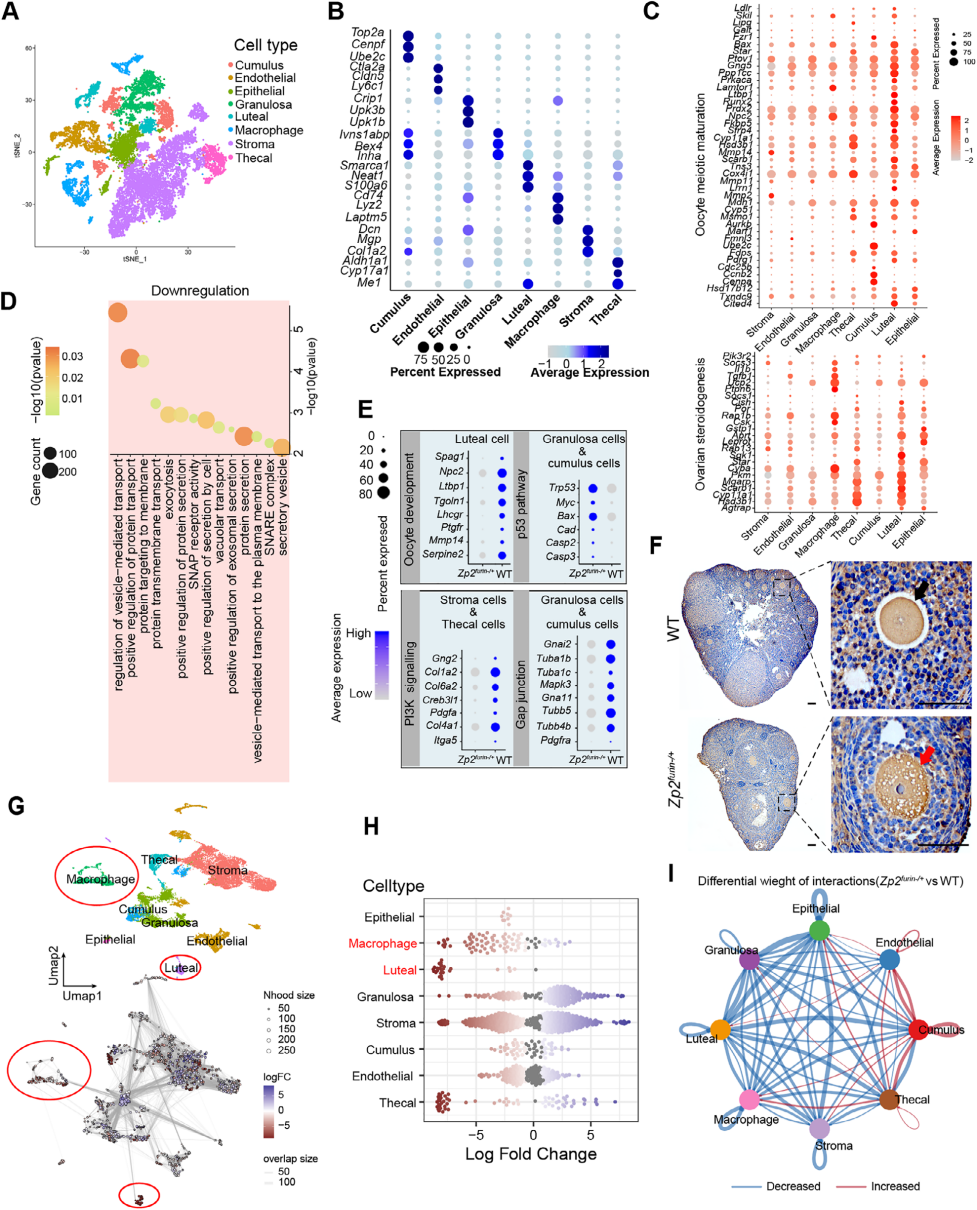
Pathological signatures of EFS determined through single‐cell RNA‐sequencing profiling of ovaries from *Zp2^furin−/+^* mice. (A) *t*‐SNE plot showed eight ovarian cell types. (B) Dot plot illustrating expression of signature genes across eight major ovarian cell types, including stromal, endothelial, thecal, granulosa, cumulus, luteal, epithelial cells, and macrophages. Dot diameter reflects the proportion of cells expressing each gene, whereas color intensity denotes the mean expression level. (C) Dot plot showing the expression of genes related to oocyte meiotic progression and ovarian steroidogenesis across ovarian cell types. Dot diameter reflects the proportion of cells expressing each gene, whereas color intensity denotes the mean expression level. (D) Representative GO analysis of downregulated differentially expressed genes (DEGs) between the WT and *Zp2^furin−^* ^/+^ groups showing decreased activity in transmembrane transport‐related processes. (E) Dot plot showed the DEGs in different ovarian cells from WT mice and *Zp2^furin−/+^* mice. (F) Immunohistochemistry analyses of connexin 37 in the ovaries of *Zp2^furin−/+^* and WT mice. Black arrow denotes the normal connexin 37 staining at the surface of oocytes. Red arrow denotes the defective staining of connexin 37. Scale bars, 150 µm. (G) UMAP (top) of all cells colored by annotated cell types, accompanied by the Milo neighborhood graph (bottom) in which each node represents a neighborhood (node size: cells per neighborhood; node color: log fold change of *Zp2^furin−/+^* vs. WT). (H) Neighborhood log fold changes grouped by cell type (each dot = one neighborhood). (I) Circle plot illustrating changes in ligand–receptor interaction strength among ovarian cell types in *Zp2^furin−/+^* mice compared with WT. Blue edges indicate decreased signaling, and red edges indicate increased signaling. ZP, zona pellucida.

Next, we performed single‐cell RNA sequencing to characterize cell pathologic features within ovaries from EFS mice. In total, 23 clusters were identified across WT and *Zp2^furin−^* ^/+^ ovary (Figure S5A). We annotated eight major cell types based on signature genes: stromal cells (*Dcn*, *Col1a2*, and *Mgp*), endothelial cells (*Cldn5* and *Ly6c1*), thecal cells (*Aldh1a1*), granulosa cells (*Bex* and *Lnha*), cumulus cells (*Top2a*, *Cenpf*, and *Ube2c*), luteal cells (*Neat1* and *Smarca1*), epithelial cells (*Crip1*, *Upk1b*, and *Upk3b*), and macrophages (*Lyz2* and *Cd74*) (Figure 7A,B). Notably, genes implicated in oocyte meiotic maturation were enriched in granulosa, cumulus, luteal, and thecal cells, with luteal and thecal cells were also prominently associated with ovarian steroidogenesis (Figure 7C).

GO analysis showed that upregulated differentially expressed genes (DEGs) in the *Zp2^furin−^* ^/+^ ovaries were enriched for “positive regulation of apoptotic process” and “negative regulation of gene expression,” whereas downregulated DEGs were enriched for “negative regulation of apoptotic signaling pathway,” “response to hormone,” and “protein transmembrane transport/protein exosomal secretion” (Figure S5B). Strikingly, most of the downregulated networks in *Zp2^furin−^* ^/+^ group were related to protein transmembrane transport processes, consistent with incomplete exocytosis of mutant ZP2 lacking the furin site (Figure 7D). We therefore focused on the reduced expression of SNARE complex proteins, which are responsible for protein transport [38]. SNARE proteins have also been suggested to play critical roles in cell survival and apoptosis [39, 40, 41, 42, 43]; thus, we hypothesized that the impaired expression of *SNAREs* might contribute to oocyte apoptosis. qPCR confirmed *SNAREs* downregulation in *Zp2^furin−^* ^/+^ ovaries (Figure S5C).

Additionally, we analyzed the DEGs in the specific cell subpopulations. The proapoptotic molecules in the p53/Bax pathway were upregulated, and PI3K signaling which can cause oocytes to start growing and prevent apoptosis [44] was inactive in granulosa cells and cumulus cells from the *Zp2^furin−^* ^/+^group (Figure 7E). The decreased expression of SNARE proteins has been suggested to induce apoptosis in p53 and PI3K pathways [40, 41, 42, 43]; we thus inferred that the reduced expression of SNARE complex proteins might induce oocyte apoptosis via the p53 and PI3K pathways. Moreover, granulosa and cumulus cells in the *Zp2^furin−^* ^/+^ group showed reduced expression of gap‐junction‐related genes (*Gnai2*, *Tuba1b*, *Tuba1c*, *Mapk3*, *Gna11*, *Tubb5*, *Tubb4b*, and *Pdgfra*), suggesting impaired granulosa‐oocyte metabolic coupling [39] (Figure 7E). Immunohistochemistry for connexin 37 confirmed the defective gap junctions in *Zp2^furin−/+^* mice (Figure 7F).

**FIGURE 8:**
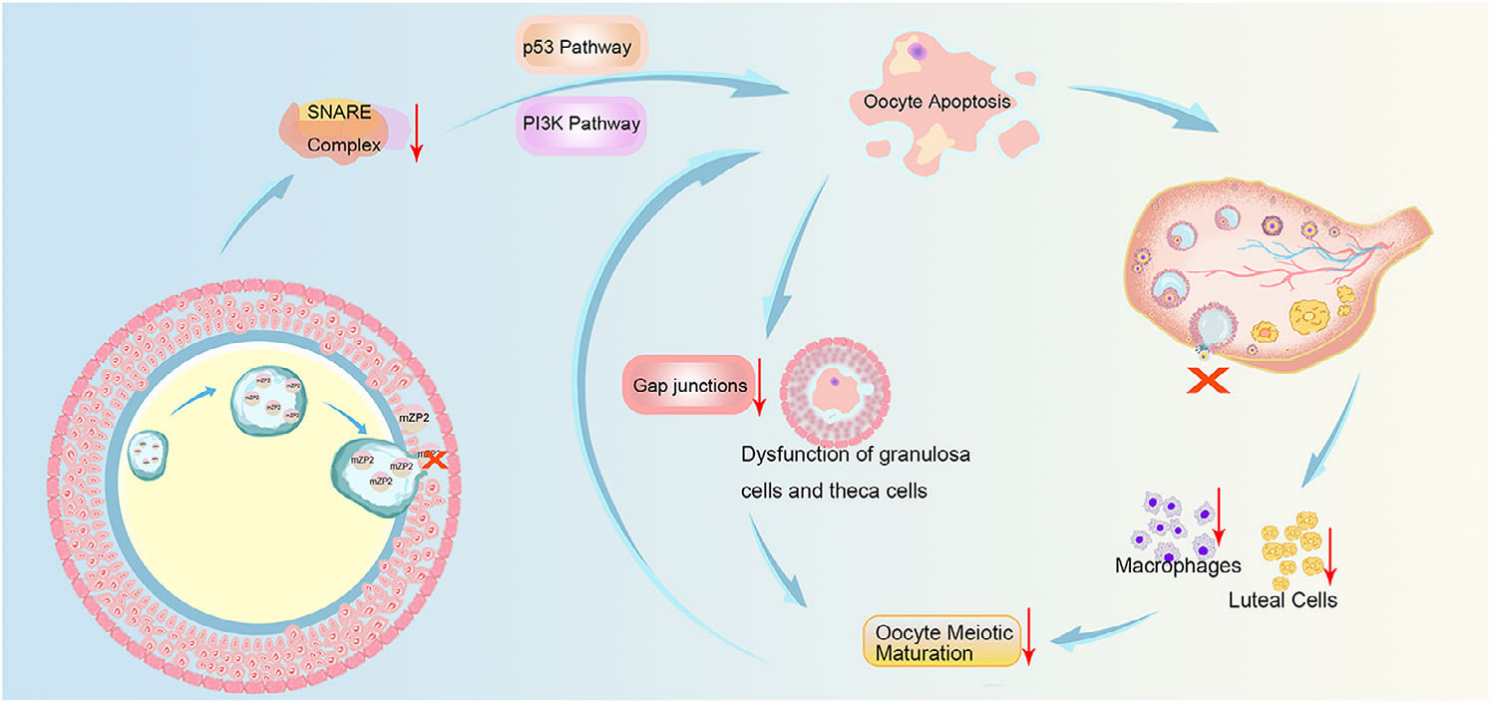
The proposed model for ZP2 protein without furin site inducing EFS. The furin cleavage site mutation leads to a failure in transmembrane transport process of non‐modified ZP2 protein. This unsuccessful ZP2 secretion might result in the decreased expression of exocytosis complex SNAREs, which further induces oocyte apoptosis via p53 pathway and PI3K signaling. This oocyte apoptosis process consequently brings out the dysfunction of critical networks essential for oocyte development, which aggravates oocyte apoptosis and eventually leads to EFS. mZP2, mutant ZP2.

Cell composition analysis revealed a marked reduction of luteal cells in the *Zp2^furin−/+^* group (Figure 7G,H), consistent with ovulatory abnormalities. With diminished luteal cells, luteal‐enriched genes essential for oocyte development, including *Lhcgr*, *Spag1*, *Npc2*, *Ltbp1*, *Tgoln1*, *Serpine2*, *Mmp14*, *Admts1*, and *Ptgfr*, were markedly downregulated in the *Zp2^furin−/+^* group relative to the WT group [45, 46, 47, 48, 49, 50, 51] (Figure 7E,G, and H). Importantly, the numbers of macrophages, whose formation and increase mainly depend on the luteal phase, were also reduced in the *Zp2^furin−^* ^/+^ group [47] (Figure 7G,H). It has been recognized that macrophages, which produce diverse growth factors and cytokines to promote follicular growth and suppress cell apoptosis during oogenesis, play indispensable roles in ovarian events [52]. Thus, the substantially reduced numbers of macrophages aggravate the disruption of normal oocyte development. Subsequently, we profiled intercellular communication across ovarian cell types and observed a generalized decline of ligand–receptor signaling in most populations of the *Zp2^furin−/+^* ovary (Figure 7I). Notably, granulosa cells showed marked reductions in NPR2‐ and KIT‐mediated interactions, changes that may contribute to the pathophysiology of EFS [53, 54] (Figure S5D). In *Zp2^furin−/+^* ovary, death‐associated signaling, particularly the TWEAK and ANNEXIN‐mediated interactions, was prominently enhanced across multiple cell lineages [55, 56] (Figure S5D). However, pseudotime trajectory analysis revealed no discernible differences between WT and *Zp2^furin−/+^* ovaries (Figure S6). Collectively, single‐cell RNA‐sequencing highlights abnormal cell composition (reduced luteal cells and macrophages) and perturbed gene‐expression programs in the *Zp2^furin−^* ^/+^ ovary, which contribute to the pathogenesis of EFS. We propose that failed furin‐mediated cleavage of ZP2 impairs ZP2 secretion and depresses SNARE‐dependent exocytosis, activating apoptotic signaling (p53/PI3K) and inducing oocyte apoptosis. Concomitant loss of ovulation reduces luteal cells and macrophages, further compromising the ovarian microenvironment and oocyte support, ultimately converging to produce EFS (Figure 8).

## 3 Discussion

Our study comprehensively revealed that furin‐mediated cleavage of ZP proteins is indispensable for female reproduction. Notably, impairment of furin cleavage in ZP2 causes female infertility associated with EFS in a dominant inheritance pattern, which is distinct from the previously reported thin‐ZP or free‐ZP oocytes resulting from biallelic loss‐of‐function mutations of *Zp2* under a recessive inheritance pattern. Strikingly, disruption of furin‐mediated cleavage modification in ZP1 and ZP3 also affected ZP formation in a subset of oocytes, resulting in reduced fecundity in mouse models. Mechanistically, we illustrated that abnormal PTM of ZP2 due to defective furin cleavage impairs its transmembrane transport. The consequent decrease in SNARE complex proteins might induce oocyte apoptosis through activation p53 pathway and inhibiting PI3K signaling, thereby disturbing the regulatory networks essential for oocyte development and ultimately causing EFS.

Furin‐mediated cleavage is a common PTM involved in various physiological and pathological events. However, as far as we are aware, the role of this type of PTM in ZP proteins during female reproduction has rarely been reported. Importantly, the generation of mouse models lacking furin‐mediated cleavage of ZP proteins enabled us to reveal the critical importance of this PTM for oocyte formation. We identified female mice carrying a heterozygous cleavage‐resistant variant at the ZP2 furin site that exhibited EFS phenotypes, revealing the essential role of furin‐mediated cleavage of ZP2 in oocyte development. Moreover, impeding furin‐mediated cleavage in ZP1 and ZP3 in mouse models resulted in mildly reduced fertility, likely due to partial interference with the transmembrane transport of ZP1 and ZP3, which resulted in defective ZP in a subset of oocytes. Although the overall abundance of ZP proteins has been recognized as a key determinant of oocyte formation, our work reveals that the PTM of ZP proteins is also linked to oocyte development, suggesting that the functional impact of PTM should not be ignored in the investigation of infertility etiology.

To date, limited research has investigated whether furin cleavage site is associated with the release of ZP proteins. In vitro studies have reported contradictory findings regarding the role of furin‐mediated cleavage in the extracellular transport of ZP3 [16, 17, 18, 19, 20]. An in vivo observation further revealed that ZP3 secretion was not entirely abolished in transgenic mice harboring a furin‐site mutation [57]. Importantly, in our study, we generated mouse models harboring an abolished ZP3 furin site (RNRR→RNRS) using CRISPR‐Cas9 and found that membrane transport of the ZP3 protein was prevented in *Zp3^furin−^* ^/+^ female mice, resulting in defective ZP in a subset of oocytes. These findings suggested that the furin cleavage site is crucial for ZP3 secretion. However, the functions of the furin cleavage sites in ZP1 and ZP2 remain poorly understood. In this study, both in vitro and in vivo experiments demonstrated that the furin variant impedes ZP2 trafficking within growing oocytes. Simultaneously, our further observations in *Zp1^furin−/+^* mouse model revealed that the membrane transport of ZP1 is partly dependent on its furin cleavage site. Collectively, our study demonstrates that the furin cleavage site is imperative for the ZP proteins secretion and mutations disrupting the furin site could impair oocyte development causing female infertility.

EFS is a severe phenotype of female infertility, marked by the failure to obtain oocytes from preovulatory follicles after ovarian hyperstimulation [58], and it may not be recovered by intracytoplasmic sperm injection due to the lack of available oocytes. However, the mechanisms driving EFS are still poorly understood. Strikingly, in our study, we identified that the loss of furin‐mediated cleavage of ZP2 represents an important cause of EFS. Furthermore, single‐cell RNA‐sequencing revealed that defective extracellular transport of non‐cleavable ZP2 activates oocyte apoptotic pathways through the downregulation of SNARE complex protein. Our findings discovered a novel etiology and also a thorough molecular mechanism of EFS, which is useful for the future research of the treatment for EFS. Prior studies showed that ASTL, a member of the astacin family, targets a specific cleavage site on ZP2 [59]. In our study, we found that overexpressing ASTL in CHO‐K1 cells and in mouse oocytes reduced the cytoplasmic retention of mutant ZP2 caused by defective furin‐mediated cleavage. Moreover, literature has reported that astaxanthin can promote the expression of ASTL [25, 26]. Interestingly, our in vivo findings showed that continuous gastric perfusion of astaxanthin in *Zp2^furin−/+^* females was associated with increased ASTL abundance and partially rescued the EFS phenotype by reducing the cytoplasmic retention of mutant ZP2 and improving ZP production. Astaxanthin has been widely used in humans, and prior clinical studies have suggested it can ameliorate poor ovarian response and promote oocyte in vitro maturation by reducing oxidative stress [60, 61]. However, to our knowledge, no clinical trials have directly evaluated astaxanthin in human EFS. Therefore, our observation may have clinical relevance, but clinical use will depend on further prospective trials.

There are still some limitations in this study. Our findings were primarily based on mouse models, which may limit direct extrapolation to humans. Although astaxanthin has been widely applied in humans, its efficacy and precise mechanism in rescuing EFS remain to be fully elucidated. Future studies employing human‐derived oocytes or ovarian organoid systems, together with clinical investigations, will be necessary to validate and extend these findings.

In conclusion, we thoroughly analyzed the functional roles of furin‐mediated cleavage of ZP proteins in female fertility, revealing that suppression of furin cleavage mediated‐PTM in ZP2 is a novel cause of EFS in mice, and anomalies of furin cleavage of ZP1 and ZP3 are associated with aberrant oocyte ZPs, impairing normal offspring production. The main mechanism underlying EFS was elucidated in this study, providing a potential target for therapeutic intervention of this disease. Therefore, focusing on the gene variations that interfere with protein PTM will provide novel perspectives for the molecular diagnosis of female infertility in clinical practice.

## 4 Materials and Methods

### 4.1 Animal Models

All procedures involving animals followed institutional guidelines and national regulations for laboratory animal welfare. The experimental protocols of animal were approved by the Experimental Animal Management and Ethics Committee of West China Second University Hospital, Sichuan University (approval no. 2021033). Mammalian ZP proteins carry a conserved furin cleavage motif (RX(K/R)R) [15]. In mouse, this conserved furin cleavage motif spans ZP1 aa 545–548, ZP2 aa 632–635, and ZP3 aa 350–353 [62]. We therefore mutated the invariant Arg residues within the CFCS spans (e.g., ZP1 Arg548, ZP2 Arg632, and ZP3 Arg353) to disrupt furin cleavage. The mouse strains *Zp1* ^Arg548Gln/+^ (*Zp1^furin−/+^*), *Zp2* ^Arg632Ser/+^ (*Zp2^furin−/+^*), and *Zp3* ^Arg353Ser/+^ (*Zp3^furin−/+^*) were established using CRISPR–Cas9–based genome editing [63]. Briefly, guide RNAs (gRNAs) were designed to target exon 11 of *Zp1* (gRNA1), exon 16 of *Zp2* (gRNA2), and exon 7 of *Zp3* (gRNA3) to generate the corresponding *Zp1* ^Arg548Gln/+^, *Zp2* ^Arg632Ser/+^ mice, and *Zp3* ^Arg353Ser/+^ mice. The gRNAs were synthesized with the HiScribe T7 High Yield RNA system (E2050s, NEB) and subsequently purified using MEGAclear Kit (AM1908, Invitrogen) in vitro. Single‐strand oligodeoxynucleotides (ssODNs) with a corresponding mutation and a synonymous mutation at the protospacer adjacent motif were synthesized, respectively, by Sangon Biotech (ssODN1: c.G1643A for *Zp1* ^Arg548Gln/+^; ssODN2: c.G1896C for *Zp2* ^Arg632Ser/+^; ssODN3: c.G1059C for *Zp3* ^Arg353Ser/+^). The ssODN1/gRNA1/Cas9 mRNAs, ssODN2/gRNA2/Cas9 mRNAs, and ssODN3/gRNA3/Cas9 mRNAs were microinjected separately into B6D2F1 (C57BL/6×DBA/2J) zygotes. Genotyping of the resulting pups was performed by Sanger sequencing. The founder mice carrying heterozygous for a missense mutation in *Zp1*, *Zp2*, or *Zp3* (*Zp1* ^Arg548Gln/+^, *Zp2* ^Arg632Ser/+^, or *Zp3* ^Arg353Ser/+^) were subsequently backcrossed onto the C57BL/6 background for at least two generations, and the heterozygous offspring were used for subsequent experiments. The sequences of ssODNs, gRNAs, and genotyping primers are provided in Table S2.

### 4.2 Single‐Cell RNA Sequencing

The ovarian samples from 12‐week‐old female mice were immersed in M2 medium (M7167, Sigma‐Aldrich). Then, the samples were minced and digested at 37°C for 30 min in complete media containing collagenase type IV (17104‐019, Invitrogen, Waltham, MA, USA), DNase I (260913, Merck), and hyaluronidase V (bs‐1235P, Bioss). The resulting cell suspension was passed through a 70‐µm strainer, washed twice with PBS, treated with Red Blood Cell Lysis Buffer (C03‐05002, Bioss), and subsequently resuspended in PBS. Using Chromium Single Cell 5′ Library and Gel Bead Kit (1000006, 10x Genomics) together with Chromium Single Cell A Chip Kit (120236, 10x Genomics), the cell suspension (300–600 living cells per microliter determined by Count Star) was loaded onto the Chromium single cell controller (10x Genomics) to generate single‐cell gel beads in the emulsion. Libraries were prepared with Single Cell 5′ Library and Gel Bead Kit and sequenced on an Illumina Novaseq 6000 platform using PE150 mode, achieving a depth of no less than 77,618 reads per cell (Novogene, Beijing).

Raw sequencing data were processed with Cell Ranger (10x Genomics). The count pipeline was used for alignment, quality filtering, barcode and UMI counting, and generation of the feature–barcode matrix. Dimensionality reduction was performed by PCA using the IRLBA algorithm, and the reduced components were embedded in two‐dimensional space with *t*‐SNE. DEGs between cell clusters were identified using the sSeq method implemented in Cell Ranger. Functional enrichment of marker genes, including GO and KEGG analysis, was conducted using the clusterProfiler R package. The primers for validation are listed in Table S3.

### 4.3 Proteomics Analysis

The primary experimental procedures for timsTOF Pro label‐free quantitative proteomics analysis include protein extraction, trypsin digestion, LC–MS/MS analysis, and database search (PTM Bio). Briefly, proteins were extracted from mouse ovaries using a lysis buffer containing 8 M urea and protease inhibitors on ice. The lysates were centrifuged at 14,000 × *g* for 15 min, and the clarified supernatants were collected and quantified by the BCA assay. A total of 100 µg of protein from each sample was digested with trypsin (1:50, w/w) overnight at 37°C. The resulting peptides were desalted, dried, and subsequently analyzed on a timsTOF Pro mass spectrometer (Bruker) and a nanoElute LC system in DDA‐PASEF mode. The resulting spectra were searched against the *Mus musculus* UniProt database in MaxQuant to enable protein identification and quantification, using a false discovery rate threshold of <1%. The data were subsequently normalized, missing values were imputed, and statistical analyses were performed. Proteins showing a fold change of ≥1.2 were considered differentially expressed.

### 4.4 Expression Plasmids

Expression plasmids encoding wild‐type *ZP1/ZP2/ZP3/ZP4* (pENTER‐*ZP1/ZP4*, pcDNA3.1‐*ZP3*, and pDEST‐*ZP2*) and pReceiver‐M35‐*ASTL* were obtained from Vigene Biosciences (Jinan, China). The furin‐cleavage‐site mutation (c.1917G>C, p.R639S) was introduced into WT‐*ZP2* plasmid using the KOD‐Plus‐Mutagenesis Kit (SMK‐101, Toyobo) following the manufacturer's instructions. Antibodies used for western blotting, co‐immunoprecipitation (Co‐IP), and immunofluorescence staining are listed in Table S4.

### 4.5 Co‐IP and Immunoblotting

The cultured cells and mouse ovarian samples were lysed in lysis buffer (pp1802, BioTeke) supplemented with a protease inhibitor cocktail (04693159001, Roche) for 30 min at 4°C. Equal amounts of protein were resolved by sodium dodecyl sulfate–polyacrylamide gel electrophoresis (SDS‐PAGE) and transferred onto polyvinylidene fluoride membranes (ISEQ00010, Millipore). Signals were visualized using the Super ECL Plus Western Blotting Substrate (P1050, Applygen) and captured with a Bio‐Rad Imaging System (1708280, Bio‐Rad).

For the Co‐IP assay, protein lysates were incubated with the primary antibodies at 4°C overnight, followed by binding with 50 µL Magnetic Beads (B23202, Selleck) for 1 h at room temperature. The beads were subsequently washed three times with buffer (50 mM Tris‐HCl, pH 7.4; 0.1% Triton X‐100; 500 mM NaCl), after which the bound proteins were eluted with 1.2× SDS loading buffer and heated at 95°C for 5 min. The eluates were then subjected to SDS–PAGE and immunoblot analysis. Antibody details are listed in Table S4.

### 4.6 Immunofluorescence and H&E

For immunostaining, the ovaries and cultured cells were fixed in 4% paraformaldehyde, permeabilized with 0.5% Triton X‐100, and blocked with 1% BSA. Samples were then incubated with primary antibodies overnight at 4°C, followed by incubation with Alexa Fluor 488‐ or Alexa Fluor 594‐conjugated secondary antibodies (A‐21206 and A‐21203, Thermo Fisher) for 1 h at room temperature, and finally counterstained with 4,6‐diamidino‐2‐phenylindole (DAPI, Sigma‐Aldrich, D9542) to label the nuclei. Images were acquired using a laser scanning confocal microscope (Olympus).

For H&E staining, mouse ovarian tissues were fixed overnight in 4% paraformaldehyde, dehydrated through ethanol, embedded in paraffin, and cut into 5‐µm sections. The sections were subsequently stained with H&E for histological examination.

### 4.7 TEM Assay

For TEM analysis, ovarian tissues were fixed in 3% glutaraldehyde and 1% buffered osmium tetroxide. Samples were dehydrated through graded acetone series and embedded in Epon 812. Prior to ultrathin‐sectioning, semi‐thin slices were prepared to locate oocytes under a light microscope. Ultrathin sections were then stained with lead citrate and uranyl acetate and examined using a TEM (TECNAI G2 F20, Philips).

### 4.8 Oocyte Microinjection

GV‐stage oocytes were isolated from the ovaries of 8‐week‐old C57 female mice 24 h after pregnant mare's serum gonadotropin injection. Approximately 5–10 pL of mRNA solution (500 ng/µL) was microinjected into each oocyte using a Nikon microscope (TS2R) equipped with a three‐axis joystick and hydraulic micromanipulator (NT‐88‐V3, Nikon NARISHIGE). Following injection, the oocytes were cultured in vitro in M16 medium (Sigma‐Aldrich, M7292) containing 10% FBS for 48 h at 37°C in an atmosphere of 5% CO_2_ and then fixed for immunofluorescence.

### 4.9 Astaxanthin Administration

Three‐week‐old *Zp2^furin−/+^* female mice were administered 1 mL of astaxanthin solution (16 mg/mL) daily by gastric perfusion, with the dosage adapted from previously published studies [64, 65]. The treatment was initiated at 3 weeks of age, a prepubertal folliculogenesis window occurring before the first ovulation, when ZP proteins begin to form. Mice received daily oral administration for 3 months. After treatment, ovaries were collected for morphological and histological analyses, including H&E staining, TEM, and immunofluorescence. A 3‐month intervention spans puberty to sexual maturity, encompassing at least four to five complete follicular waves. This prolonged treatment allows comprehensive assessment of the long‐term effects of astaxanthin on the ovarian microenvironment and oocyte quality.

### 4.10 Ultra‐Performance Liquid Chromatography Analysis

Analytical method: Pharmacokinetic analysis of astaxanthin (>98% pure, Sigma‐Aldrich, A9241) was performed using UPLC (Agilent 1290) with visible spectrometric detection. A rheodyne injection system delivered 10 µL of each sample into a reverse‐phase column (2.5 µm, 2.1 × 100 mm^2^). The astaxanthin was separated under an isocratic conditions using MeOH with 0.1% HCOOH–H_2_O at a 90:10 (v/v) ratio at the start and 60:40 (v/v) after 1.5 min. The flow rate was 1.0 mL/min. Astaxanthin was detected at 475 nm by detector. This experiment used the standard curve method to measure the concentration of astaxanthin. Quality control plasma samples and calibration standards were generated from a stock solution of astaxanthin (>98% pure) in ethanol, 1.0 mg/mL. We prepared standard drug‐containing plasma with concentrations of 10–3000 ng/mL, respectively, from calibration standards, diluting with blank plasma. Then, the protein of the standard drug‐containing plasma was precipitated by ethanol. We injected the standard product and tested it using the above methods after collecting the supernatant and filtering. We recorded the chromatogram calculated the peak‐area ratio between the analyte and the internal standard. The plasma analyte concentration (*X*) was used as the *x*‐axis variable, and the corresponding peak‐area ratio (*Y*) relative to the internal standard was plotted on the *y*‐axis. A regression equation was obtained using 1/*X* weighting, yielding: *Y* = 305.19X‐7797.8, *R* ^2^ = 0.9998. The linear range was 10–3000 ng/mL.

Blood sampling: Following intragastric administration of astaxanthin, blood was collected from mice at 10 min, 30 min, 1 h, 2 h, 3 h, 4 h, 6 h, 8 h, 12 h, and 24 h. Eyeball blood was drawn into heparin‐coated centrifuge tubes, centrifuged at 3000 rpm for 10 min, and the supernatant was harvested. After that, we precipitated the protein in plasma with ethanol, collected supernatant, and filtered, ready for injection. According to “analytical method,” we collected the retention time and peak area and substituted into the obtained standard curve and linear range formula to calculate the plasma concentration.

Pharmacokinetic analysis was performed by determining key parameters for astaxanthin: *C*_max_ (maximum observed plasma concentration), *t*_max_ (the time to reach maximum concentration), AUC_(0→∞)_(area under the curve calculated to infinity), and *t*_1/2_ (elimination half‐life). *C*_max_ and *t*_max_ were directly read from the obtained drug‐time curve. AUC _(0→∞)_ and *t*_1/2_ were calculated with statistical moment analysis.

Ovarian sampling: After collecting the ovaries of mice that were given intragastric administration for 30 min, 1 h, 1.5 h, 2 h, 2.5 h, 3 h, 4 h, and 5 h and freezing ground with ethanol, we centrifuged (3000 rpm, 10 min) it and took the supernatant. Then, we repeated the centrifugation operation after ethanol precipitation of the protein, took the supernatant, and filtered. Similarly, according to analytical method, the concentration of astaxanthin in the ovaries was detected.

### 4.11 Statistics

Statistical analyses were carried out with the SPSS software (version 17.0; IBM Corporation, USA). Results are reported as the mean ± SEM. Differences between experimental and control groups were evaluated using Student's *t*‐test. A *p* values below 0.05 was regarded as statistically significant.

## Author Contributions

Ying Shen designed and supervised the study experiments. Ying Shen, Tiechao Ruan, Xiang Wang, and Xueguang Zhang conducted experiments and analyzed most of the data. Yan Wang collected data. Zhenbo Wang generated the CRISPR‐Cas9 mice. Chuan Jiang, Sixian Wu, and Yunchuan Tian carried out cell experiments. Xinyao Tang, Jun Ma, Shikun Zhao, and Liangchai Zhuo undertook the immunofluorescence staining. Mohan Liu and Siyu Dai performed qPCR. Ying Shen wrote the manuscript with input from others. Ying Shen and Wenming Xu provided financial support and valuable suggestions. All authors have read and approved the final manuscript.

## Funding

This study was supported by National Key Research and Development Program of China (2018YFC1002804), National Natural Science Foundation of China (82471650) and Sichuan Science and Technology Program (2024YFFK0267).

## Ethics Statement

The animal experiments were approved by the Experimental Animal Management and Ethics Committee of West China Second University Hospital, Sichuan University (approval no. 2021033). All animal procedures complied with the Animal Care and Use Committee of Sichuan University.

## Conflicts of Interest

The authors declare no conflicts of interest.

## Supporting information




**Figure S1**: Generation and phenotypic assessment of *Zp2furin* *^−^* */+* mice. (A) The genotypes of WT mice (*n* = 3) and *Zp2furin^−^/+* (*Zp2* c.1896G>C) mice (*n* = 3). The wild‐type site was indicated by a black arrow. The heterozygous mutation site was indicated by a red arrow. (B) The ovary sizes of *Zp2furin^−^/+* mice (*n* = 6) were less than those of WT mice (*n* = 6). Student's *t*‐test; *p < 0.05; error bars, s.e.m. (C) Statistical analysis of zona pellucida (ZP) thickness measured from transmission electron microscopy (TEM) images in WT (*n* = 3) and *Zp2furin^−^/+* female mice (*n* = 3). Student's *t*‐test; *p < 0.05; error bars, s.e.m. (D–F) Quantitative analysis of immunofluorescence intensity using ImageJ software (version 1.54r) showed increased cytoplasmic retention of ZP2(D), ZP1 (E), and ZP3 (F) proteins in primary, secondary, and tertiary oocytes from *Zp2furin^−^/+* female mice (*n* = 3) compared with WT controls (*n* = 3). Student's *t*‐test; *p < 0.05; error bars, s.e.m.
**Figure S2**: Construction of mice with heterozygous mutations at furin cleavage sites of *Zp1* and *Zp3*. (A and B) The schematic illustration of the targeting strategy for generating *Zp1furin^−^/+* (A; *Zp1* c.1643G>A) and *Zp3furin^−^/+*(B; *Zp3* c.1059G>C) mice. (C and D) Sanger sequence chromatograms of the genotypes of *Zp1furin^−^/+* (C) and *Zp3furin^−^/+* (D) female mice. The wild‐type site is indicated by a black arrow. Red arrows denote the heterozygous mutation sites. Results are representative of three independent experiments. (E and F) The weight of ovaries from *Zp1furin^−^/+* (*n* = 6) (E) and *Zp furin^−^/+* mice (*n* = 6) (F) was similar with that of WT mice (*n* = 6). Student's *t*‐test; NS, not significant; error bars, s.e.m. (G and H) The numbers of ovarian follicles at different developmental stages between WT (*n* = 3) and *Zp1furin^−^/+* mice (*n* = 3) (G) or *Zp3furin^−^/+* mice (*n* = 3) (H). Student's *t*‐test; NS, not significant; error bars, s.e.m. (I) Statistical analysis of zona pellucida (ZP) thickness measured from transmission electron microscopy (TEM) images in WT (*n* = 3), *Zp3furin−/+* (n = 3) and *Zp3furin−/+* (n = 3) female mice. Student's *t*‐test; *p < 0.05; error bars, s.e.m.
**Figure S3**: The effects of lacking furin cleavage modification on ZP proteins. (A) Immunofluorescence staining showed that WT‐ZP2 and ZP2*furin^−^/^−^* shared the similar colocation with endoplasmic reticulum (ER), GM130 (Golgi), and STX6 (multivesicular bodies). Green, ZP2; red, ER/GM130/STX6; blue, DAPI; scale bars, 10 µm. Results are representative of three independent experiments. (B) Immunofluorescence staining represented the altered localization of WT‐ZP2 when co‐expressed with the mutant ZP2. Green, HA; red, Myc; blue, DAPI; scale bars, 10 µm. Results are representative of three independent experiments. (C and D) Immunofluorescence staining showed the abnormal accumulation of ZP1 (C) and ZP3 (D) in several CHO cells transfected with *ZP1furin^−^/^−^* and *ZP3furin−/−* plasmids. Green, ZP1 or ZP3; blue, DAPI; scale bars, 10 µm. Results are representative of three independent experiments.
**Figure S4**: The differential proteins expressed in WT and *Zp2furin* *^−^* */+* mice. (A) Volcano plot showing the distribution of proteins identified between *Zp2furin^−^/+* and WT ovaries. The dashed lines indicate the thresholds for differential expressions. (B and C) The significantly altered key proteins involved in oocyte development were further confirmed in ovaries of *Zp2furin^−^/+* mice (*n* = 3) by immunofluorescence staining (B) and western blotting (C). Green, LHCGR/FDPS/YTHDC1/MAP3K5/UBE2C/DAPK3/KIF1B; blue, DAPI; scale bars, 600 µm.
**Figure S5**: Dysregulation of SNARE complex genes and intercellular signaling in *Zp2furin* *^−^* */+* ovaries (A) *t*‐SNE visualization of mouse ovarian single‐cell transcriptomes showing distinct cell clusters. (B) Representative GO analysis of downregulated (top) or upregulated (down) DEGs between WT group and *Zp2furin^−^/+* group. Red rectangles highlight the reduced processes involved in transmembrane transport. (C) qPCR confirmed several down regulated genes of SNARE complex. Results are representative of three independent experiments. Student's *t*‐test; *p < 0.05; error bars, s.e.m. (D) CellChat heat maps display the relative strength of ligand–receptor signaling for each pathway (rows) contributed by each cell type (columns).
**Figure S6**: Pseudotime trajectory analysis of ovarian cell populations in WT and *Zp2furin* *^−^* */+* mice. (A and B) Monocle‐based trajectory plots showing developmental pseudotime of eight major ovarian cell types in WT (A) and *Zp2furin^−^/+* (B) ovaries. Each point represents a single cell colored by cell type.
**Table S1**: Pharmacokinetic statistical data of astaxanthin.
**Table S2**: Detailed information regarding target sequences in mouse models.
**Table S3**: The qPCR primers used in the present study.
**Table S4**: Comprehensive list of antibodies employed in the study.

## Data Availability

All the necessary data to assess the conclusions in this article are available in both the main document and the Supporting Information section. The mass spectrometry–based proteomics data have been deposited in the ProteomeXchange Consortium (via the iProX partner repository) under the dataset identifier PXD070013 (https://proteomecentral.proteomexchange.org). The single‐cell RNA sequencing data have been submitted to the OMIX database (accession number OMIX012281; https://ngdc.cncb.ac.cn/omix).
