# Supplementary material for: Furin‐Mediated Cleavage of Zona Pellucida Proteins Is Essential for Oocyte Development
Source: MedComm (2020). 2025 Dec 12;6(12):e70542. doi: 10.1002/mco2.70542 (PMC12701285; doi:10.1002/mco2.70542)
Supplement: Supplementary file 1 — Figure S1: Generation and phenotypic assessment of Zp2furin − /+ mice. (A) The genotypes of WT mice (n = 3) and Zp2furin−/+ (Zp2 c.1896G>C) mice (n = 3). The wild‐type site was indicated by a black arrow. The heterozygous mutation site was indicated by a red arrow. (B) The ovary sizes of Zp2furin−/+ mice (n = 6) were less than those of WT mice (n = 6). Student's t‐test; *p < 0.05; error bars, s.e.m. (C) Statistical analysis of zona pellucida (ZP) thickness measured from transmission electron microscopy (TEM) images in WT (n = 3) and Zp2furin−/+ female mice (n = 3). Student's t‐test; *p < 0.05; error bars, s.e.m. (D–F) Quantitative analysis of immunofluorescence intensity using ImageJ software (version 1.54r) showed increased cytoplasmic retention of ZP2(D), ZP1 (E), and ZP3 (F) proteins in primary, secondary, and tertiary oocytes from Zp2furin−/+ female mice (n = 3) compared with WT controls (n = 3). Student's t‐test; *p < 0.05; error bars, s.e.m. Figure S2: Construction of mice with heterozygous mutations at furin cleavage sites of Zp1 and Zp3. (A and B) The schematic illustration of the targeting strategy for generating Zp1furin−/+ (A; Zp1 c.1643G>A) and Zp3furin−/+(B; Zp3 c.1059G>C) mice. (C and D) Sanger sequence chromatograms of the genotypes of Zp1furin−/+ (C) and Zp3furin−/+ (D) female mice. The wild‐type site is indicated by a black arrow. Red arrows denote the heterozygous mutation sites. Results are representative of three independent experiments. (E and F) The weight of ovaries from Zp1furin−/+ (n = 6) (E) and Zp furin−/+ mice (n = 6) (F) was similar with that of WT mice (n = 6). Student's t‐test; NS, not significant; error bars, s.e.m. (G and H) The numbers of ovarian follicles at different developmental stages between WT (n = 3) and Zp1furin−/+ mice (n = 3) (G) or Zp3furin−/+ mice (n = 3) (H). Student's t‐test; NS, not significant; error bars, s.e.m. (I) Statistical analysis of zona pellucida (ZP) thickness measured from transmission electron microscopy [file MCO2-6-e70542-s001.pdf]

Furin-mediated cleavage of zona pellucida proteins is essential for oocyte development

Tiechao Ruan[1, 2#], Xiang Wang[1, #], Xueguang Zhang[1, #], Yan Wang[1, 3#], Chuan Jiang[1], Sixian Wu[1], Yunchuan Tian[1], Xinyao Tang[1], Jun Ma[1], Shikun Zhao[1], Liangchai Zhuo[1], Mohan Liu[1], Siyu Dai[1], Zhenbo Wang[4, 5, *], Wenming Xu[1, *], Ying Shen[1, 6, *]

Author Affiliations

[1] Department of Obstetrics/Gynecology, Key Laboratory of Birth Defects and Related Disease of Women and Children of MOE, West China Second University Hospital, Sichuan University, Chengdu 610041, China.

[2] Department of Pediatrics, West China Second University Hospital, Sichuan University, Chengdu 610041, China.

[3] Reproduction Medical Centre, West China Second University Hospital, Sichuan University, Chengdu 610041, China.

[4] State Key Laboratory of Stem Cell and Reproductive Biology, Institute of Zoology, Chinese Academy of Sciences, 100101 Beijing, China.

[5] University of Chinese Academy of Sciences, Beijing 100101, China.

[6] NHC Key Laboratory of Chronobiology, Sichuan University, Chengdu 610041, China.

[#] These authors contributed equally: Tiechao Ruan, Xueguang Zhang, Xiang Wang and Yan Wang.

[*] Corresponding author. Email: wangzb@ioz.ac.cn (Z.W.); xuwenming@scu.edu.cn,

(W.X.); yingcaishen01@163.com (Y.S.)

This PDF file includes:

Figure. S1 to S6

Table. S1 to S4

Original full blots

Supplementary Materials

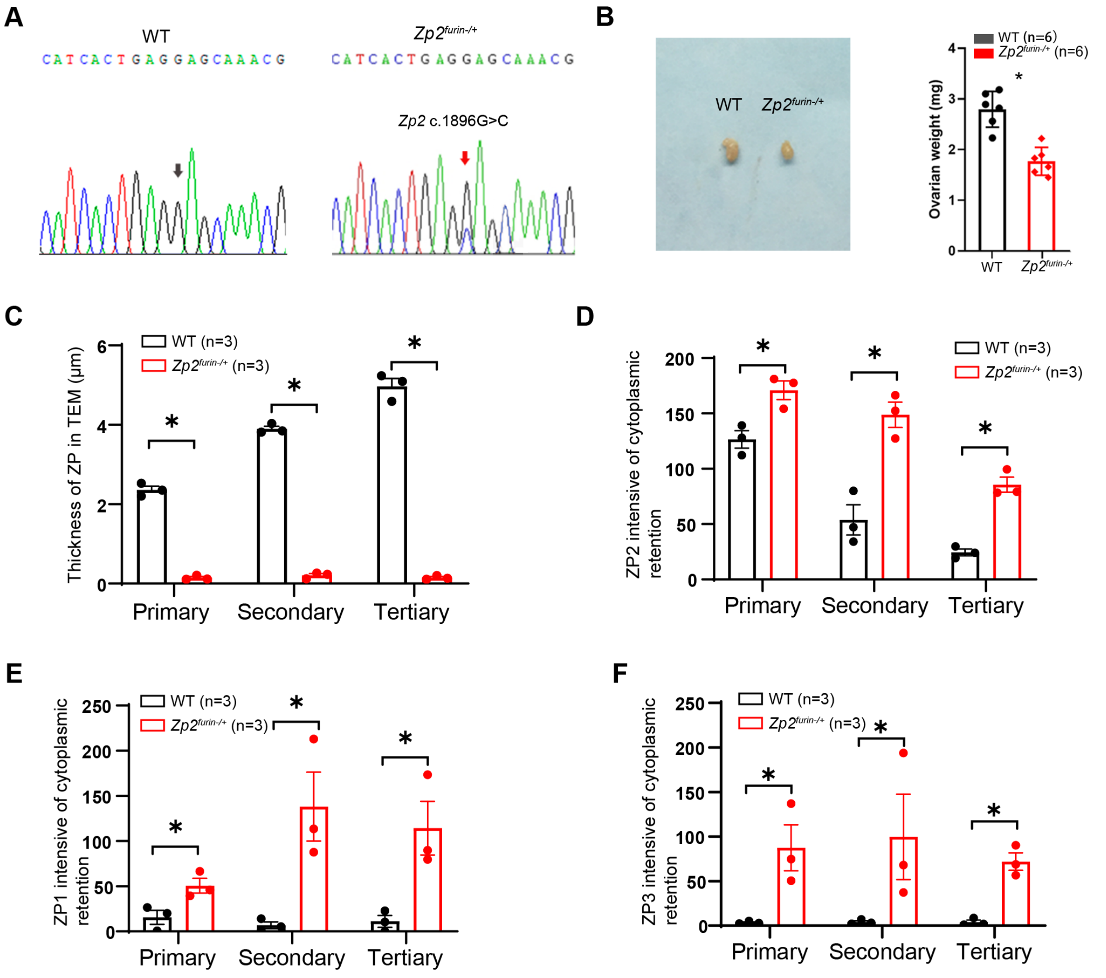

Figure S1. Generation and phenotypic assessment of *Zp2$^{furin-/+}$* mice. (A) The genotypes of WT mice (n=3) and *Zp2$^{furin-/+}$* (*Zp2* c.1896G>C) mice (n=3). The wild-type site was indicated by a black arrow. The heterozygous mutation site was indicated by a red arrow. **(B)** The ovary sizes of *Zp2$^{furin-/+}$* mice (n=6) were less than those of WT mice (n=6). Student's t-test; *p < 0.05; error bars, s.e.m. **(C)** Statistical analysis of zona pellucida (ZP) thickness measured from transmission electron microscopy (TEM) images in WT (n=3) and *Zp2$^{furin-/+}$* female mice (n=3). Student's t-test; *p < 0.05; error bars, s.e.m. **(D-F)** Quantitative analysis of immunofluorescence intensity using ImageJ software (version 1.54r) showed increased cytoplasmic retention of ZP2(D), ZP1 (E)

and ZP3 (F) proteins in primary, secondary, and tertiary oocytes from *Zp2*$^{furin-/+}$ female mice (n=3) compared with WT controls (n=3). Student's *t*-test; *$p < 0.05$; error bars, s.e.m.

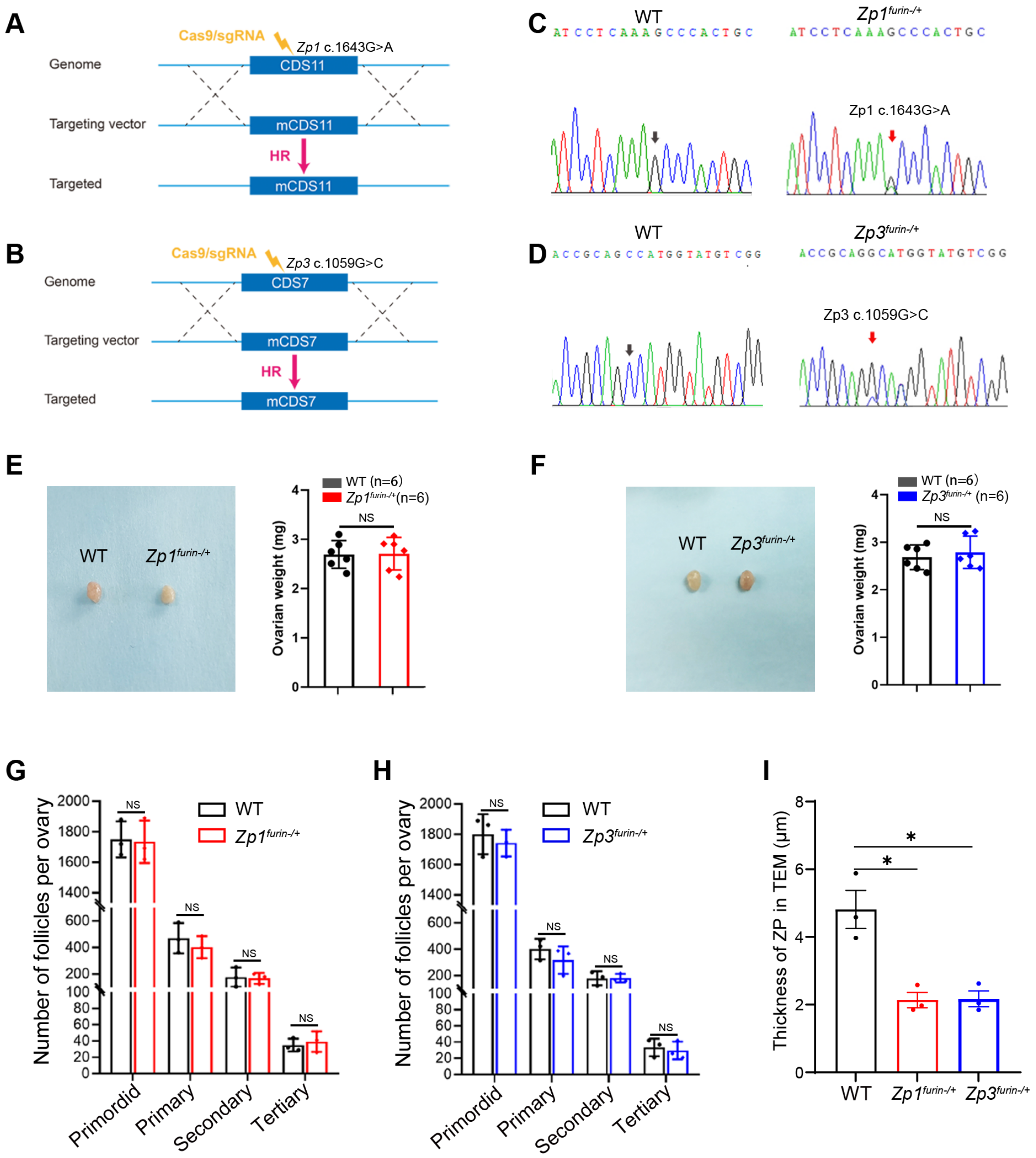

Figure S2. Construction of mice with heterozygous mutations at furin cleavage sites of *Zp1* and *Zp3*. (A and B) The schematic illustration of the targeting strategy for generating *Zp1*$^{furin–/+}$ (A; *Zp1* c.1643G>A) and *Zp3*$^{furin–/+}$(B; *Zp3* c.1059G>C) mice. **(C and D)** Sanger sequence chromatograms of the genotypes of *Zp1*$^{furin–/+}$ (C) and *Zp3*$^{furin–/+}$ (D) female mice. The wild-type site is indicated by a black arrow. Red arrows denote the heterozygous mutation sites. Results are representative of three independent experiments. **(E and F)** The weight of ovaries from *Zp1*$^{furin–/+}$ (n=6) (E) and *Zp*$^{furin–/+}$

mice (n=6) (F) was similar with that of WT mice (n=6). Student's t-test; NS, not significant; error bars, s.e.m. **(G and H)** The numbers of ovarian follicles at different developmental stages between WT (n=3) and *Zp1*$^{furin-/+}$ mice (n=3) (G) or *Zp3*$^{furin-/+}$ mice (n=3) (H). Student's t-test; NS, not significant; error bars, s.e.m. **(I)** Statistical analysis of zona pellucida (ZP) thickness measured from transmission electron microscopy (TEM) images in WT (n=3), *Zp3*$^{furin-/+}$ (n=3) and *Zp3*$^{furin-/+}$ (n=3) female mice. Student's t-test; $^{*}p < 0.05$; error bars, s.e.m.

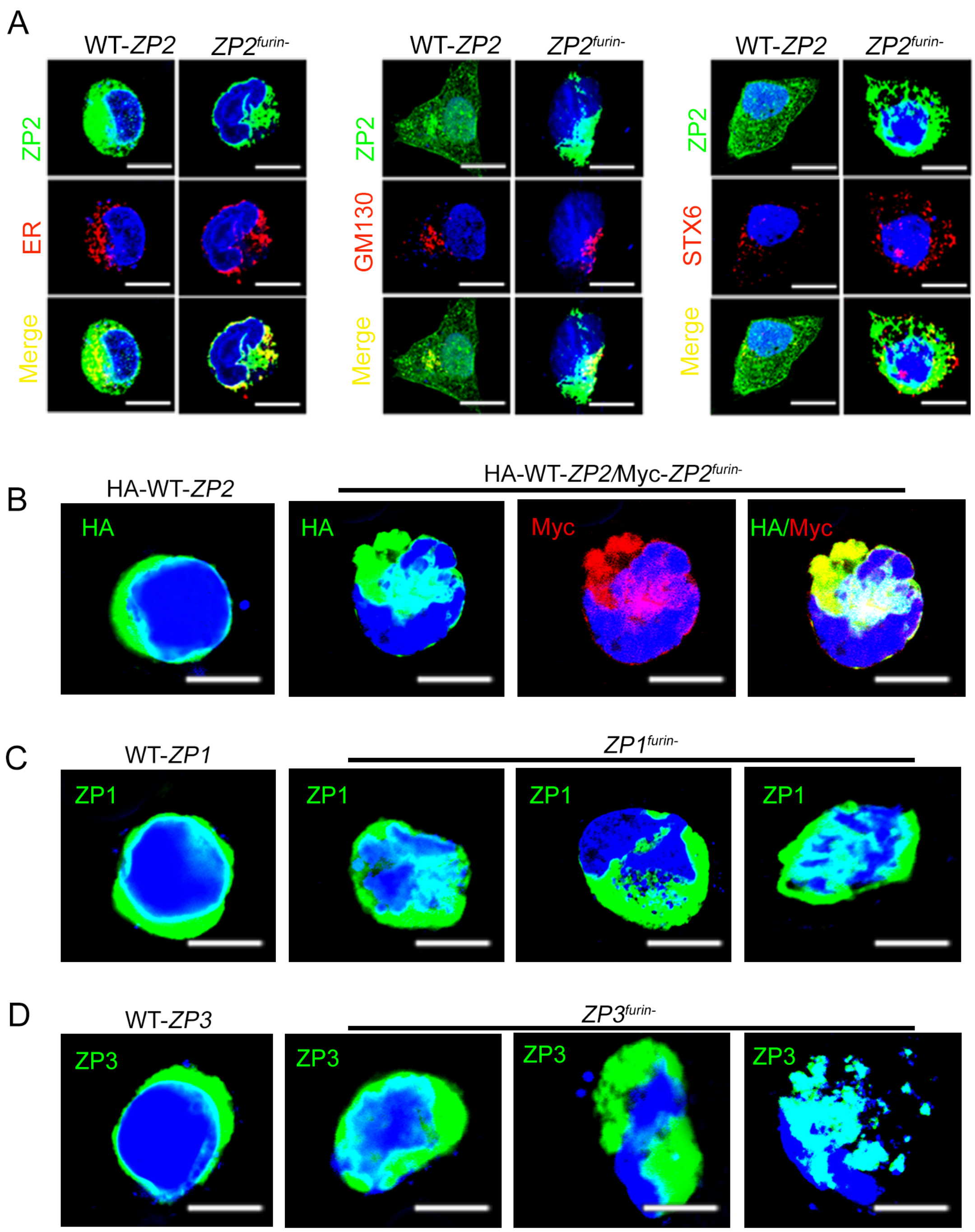

Figure S3. The effects of lacking furin cleavage modification on ZP proteins. (A) Immunofluorescence staining showed that WT-ZP2 and ZP2*furin-/-* shared the similar colocation with endoplasmic reticulum (ER), GM130 (Golgi) and STX6 (multivesicular bodies). Green, ZP2; red, ER/GM130/STX6; blue, DAPI; scale bars, 10 μm. Results are representative of three independent experiments. **(B)**

Immunofluorescence staining represented the altered localization of WT- ZP2 when co-expressed with the mutant ZP2. Green, HA; Red, Myc; blue, DAPI; scale bars, 10 μm. Results are representative of three independent experiments. **(C** and **D)** Immunofluorescence staining showed the abnormal accumulation of ZP1 (C) and ZP3 (D) in several CHO cells transfected with $ZP1^{furin-/-}$and $ZP3^{furin-/-}$ plasmids. Green, ZP1 or ZP3; blue, DAPI; scale bars, 10 μm. Results are representative of three independent experiments.

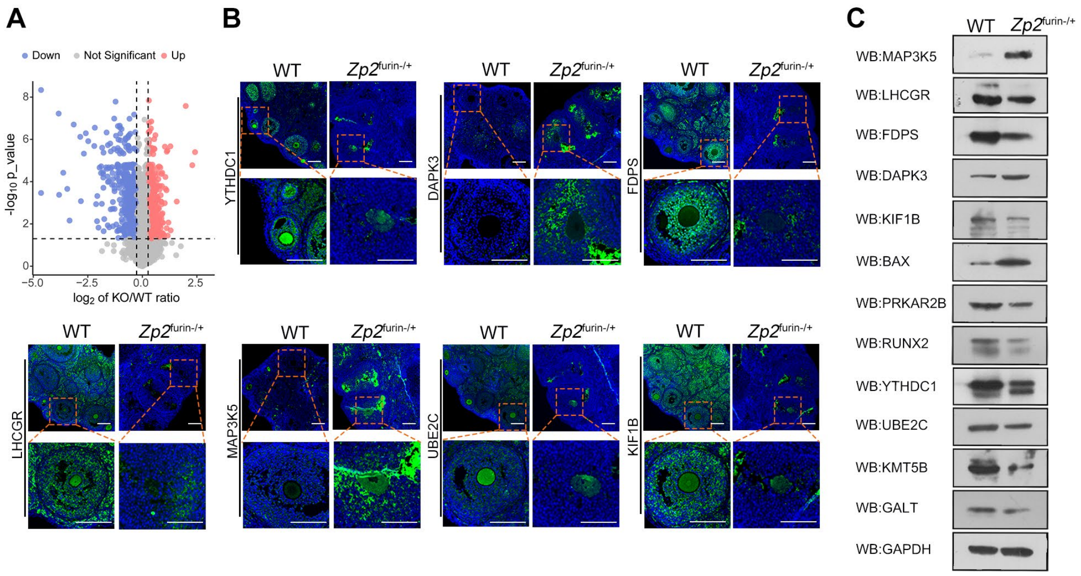

Figure S4. The differential proteins expressed in WT and *Zp2*$^{furin–/+}$ mice. (A) Volcano plot showing the distribution of proteins identified between *Zp2*$^{furin–/+}$ and WT ovaries. The dashed lines indicate the thresholds for differential expressions. **(B** and **C)** The significantly altered key proteins involved in oocyte development were further confirmed in ovaries of *Zp2*$^{furin–/+}$ mice (n=3) by immunofluorescence staining (B) and western blotting (C). Green, LHCGR/ FDPS/ YTHDC1/ MAP3K5/ UBE2C/ DAPK3/ KIF1B; blue, DAPI; scale bars, 600 μm.

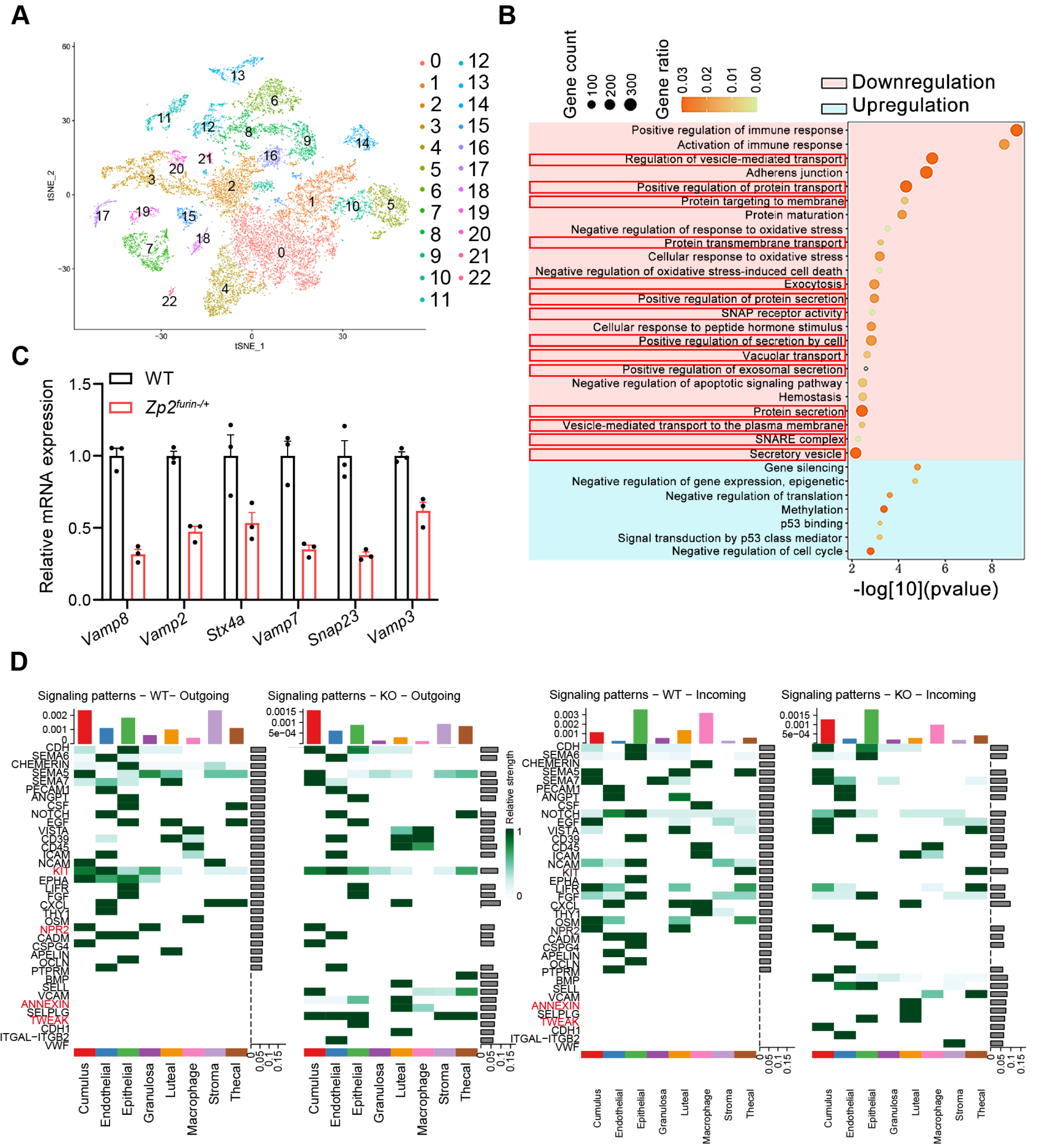

Figure S5. Dysregulation of SNARE complex genes and intercellular signaling in *Zp2*$^{furin\text{-}/+}$ ovaries (A) t-SNE visualization of mouse ovarian single-cell transcriptomes showing distinct cell clusters. **(B)** Representative GO analysis of downregulated (top) or upregulated (down) DEGs between WT group and *Zp2*$^{furin–/+}$ group. Red rectangles highlight the reduced processes involved in transmembrane transport. **(C)** qPCR confirmed several down regulated genes of SNARE complex. Results are representative of three independent experiments. Student's t-test; $*p < 0.05$; error bars,

s.e.m. **(D)** CellChat heatmaps display the relative strength of ligand–receptor signaling for each pathway (rows) contributed by each cell type (columns).

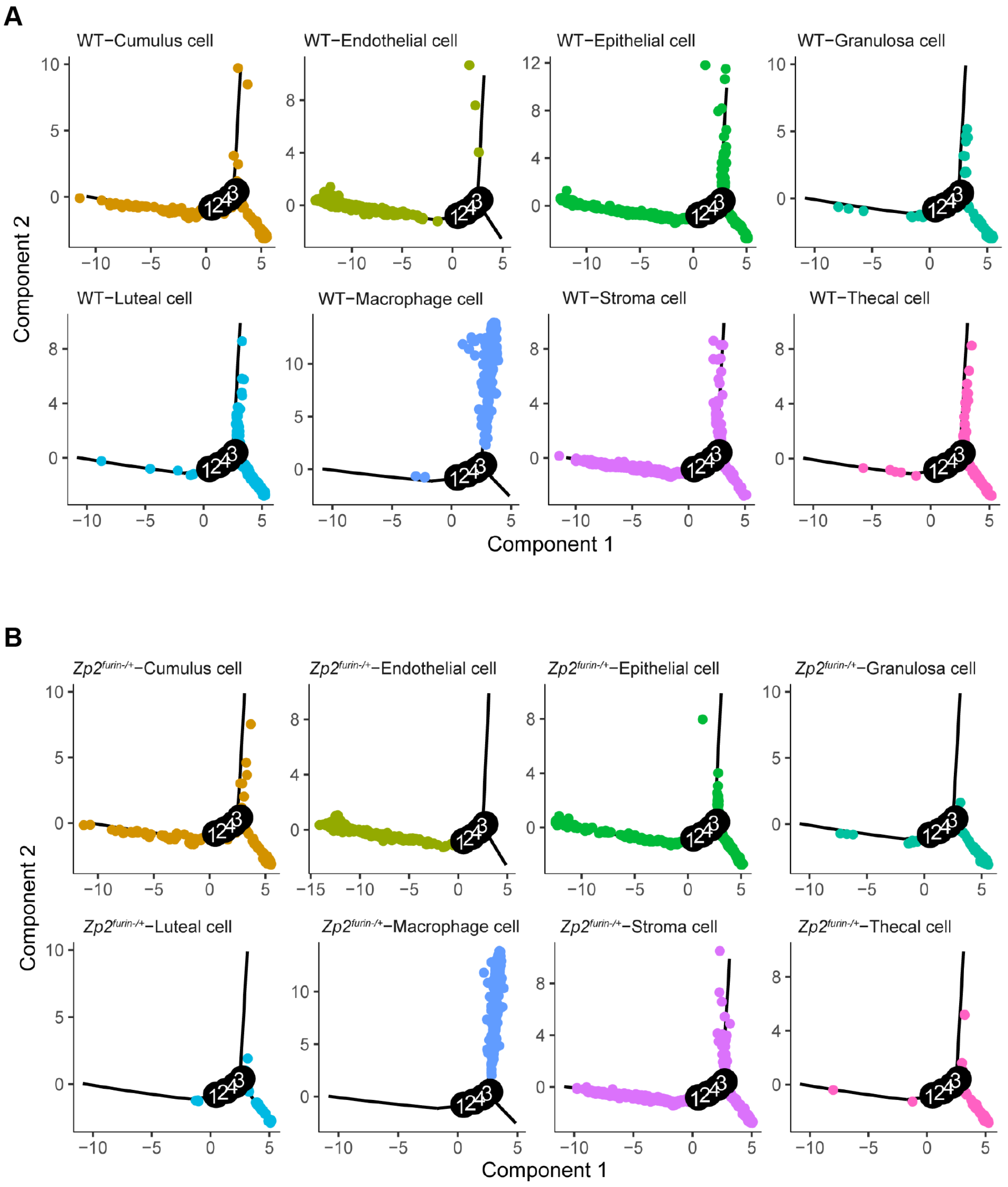

Figure S6. Pseudotime trajectory analysis of ovarian cell populations in WT and *Zp2*$^{furin-/+}$ mice. (A and B) Monocle-based trajectory plots showing developmental pseudotime of eight major ovarian cell types in WT (A) and *Zp2*$^{furin-/+}$ (B) ovaries. Each point represents a single cell colored by cell type.

Table S1. Pharmacokinetic statistical data of astaxanthin.

Parameter	Unit	1	2	3	Average
$t_{1/2}$	h	8.028316158	7.455793384	10.75696963	8.747026391
T_{max}	h	2	2	2	2
C_{max}	ng/ml	933.45	824.11	1011.23	922.93
AUC_{0-t}	ng/ml*h	3474.585	3439.1275	3932.93	3615.5475
AUC_{0-inf_obs}	ng/ml*h	3765.303539	3679.426283	4432.17709	3958.968971
$AUC_{0-t/0-inf\_obs}$		0.922790145	0.934691236	0.887358497	0.914946626
$AUMC_{0-inf\_obs}$	ng/ml*h^2	28338.53992	25908.35869	40804.01474	31683.63778
MRT_{0-inf_obs}	h	7.52622986	7.041412628	9.206314167	7.924652218

Table S2. Detailed information regarding target sequences in mouse models.

Target	Sequence (5'- 3')
gRNA1	GCAGGGCGTCGACGATCCTC
gRNA2	GCATCACTGAGGAGCAAACG
gRNA3	GTTTCTCGAAACCGCAGGCA
ssODN1	ctcttcacagttccagatccatggaccccgccagtggtccaagctagtttctcgaaaccgcagCcaTggtat gtcggggacacgggacacttattcctcctcctctttaagagctctgcttcacttcctt
ssODN2	gatctgtaaccaagtctctcttgactcccctctgtgctctgtgacttgccctgcatcactgagCagTaaacgag gtaaaaattcaatccttggtctgttgctcaaagcaagccttgattttttttttct
ssODN3	gctgactctcaggccactaggggttggaggccagacagtttttctcctgggcagggcgtcgacAatcTtct ggtcaccacaacatcactctccgggccctggatattgtaagctctccaggggcagtggg
Zp1 genotyping	F: GCCACCCCTTGACTGTGTTTCTT<br>R: GGATGACGGGCTGGGCTATT
Zp2 genotyping	F: GCAGCCCATTCCGGTCACTA<br>R: TCAAAGGATGGGTTCATCAGGCT
Zp3 genotyping	F: CACCCGGGGGCTACTCAAAT<br>R: GGCTACCCTAACCCACAAGCC

Table S3. The qPCR primers used in the present study.

Target	Forward primer (5'—3')	Reverse primer (5'—3')	Product (bp)
Vamp8	CTGCCTTGGGTGGAAACAGA	TTGTTTCGGAGGTGGTCCAG	202
Vamp2	TGAGGGTGAATGTGGACAAGG	CTTCTTAGGCAGGGCAGACT	245
Stx4a	CGACGAGTTCTTCCAGAAGGT	GCTCGACAAATTGCTGGGAC	296
Vamp7	GACAACTTACGGTTCAAGAGCA	TCTCCACGTTGAGCAACTAA ATC	195
Snap23	TGACTGTTTCTTTGAAACCAG	TGCCCACTTGAGTCAGGTTC	233
Vamp3	TGCAAGATGTGGGCGATAGG	AATGTGGGGAGGGTAGGTCA	280
Gapdh	GGTGAAGGTCGGTGTGAACG	CTCGCTCCTGGAAGATGGTG	233

Table S4. Comprehensive list of antibodies employed in the study.

Antibody or dye (application and dilution ratio)	Source	Identifier
Rabbit-Anti-ZP2 (IF,1:50)	Bioss	PB0958
Mouse-Anti-ZP1 (WB,1:500; IF,1:50)	Santa Cruz	sc-365435
Rabbit-Anti-ZP3 (WB, 1:1000; IF,1:50)	Proteintech	21279-1-AP
Rabbit-Anti-ASTL (WB, 1:300)	Proteintech	21418-1-AP
Goat-Anti-P4HB (IF,1:50)	R&D	AF4236
Mouse-Anti-GM130 (IF,1:50)	BD biosciences	610822
Rabbit-Anti-Sodium Potassium ATPase (IF,1:50)	Abcam	ab76020
Mouse-Anti-HA (WB, 1:200; IF,1:50; IP)	Santa Cruz	sc-7392
Mouse-Anti-Myc (WB, 1:1000; IF,1:1000; IP)	Cell Signaling Technology	2276s
Rabbit-Anti-Flag (WB, 1:1000; IF, 1:50; IP)	Millipore	F7425
Rabbit-Anti-MAP3K5 (WB,1:1000; IF, 1:50)	Boster	BM4220
Rabbit-Anti-LHCGR (WB,1:1000; IF, 1:50)	Proteintech	19968-1-AP
Rabbit-Anti-FDPS(WB,1:1000; IF, 1:50)	Proteintech	16129-1-AP
Rabbit-Anti- DAPK3 (WB,1:1000; IF, 1:50)	Boster	A03300
Rabbit-Anti-KIF1B (WB,1:500; IF, 1:50)	Proteintech	15263-1-AP
Rabbit-Anti-BAX (WB,1:20000; IF, 1:50)	Proteintech	50599-2-Ig
Rabbit-Anti-PRKAR2B (WB,1:1000; IF, 1:500)	Abcam	ab92312
Rabbit-Anti-RUNX2(WB,1:20000; IF, 1:1000)	Abcam	ab192256
Rabbit-Anti-YTHDC1(WB,1:1000; IF, 1:50)	Proteintech	14392-1-AP
Rabbit-Anti- UBE2C (WB,1:300; IF, 1:50)	Proteintech	12134-2-AP

Rabbit-Anti-KMT5B (WB,1:1000; IF, 1:50)	Abclonal	A15442
Rabbit-Anti-GALT(WB, 1:1000; IF, 1:50)	Abcam	ab178406
Rabbit-Anti-VASA (IF: 1:50)	Abcam	ab27591
Rabbit-Anti-CX37(IF,1:50)	Bioworld	BS6988

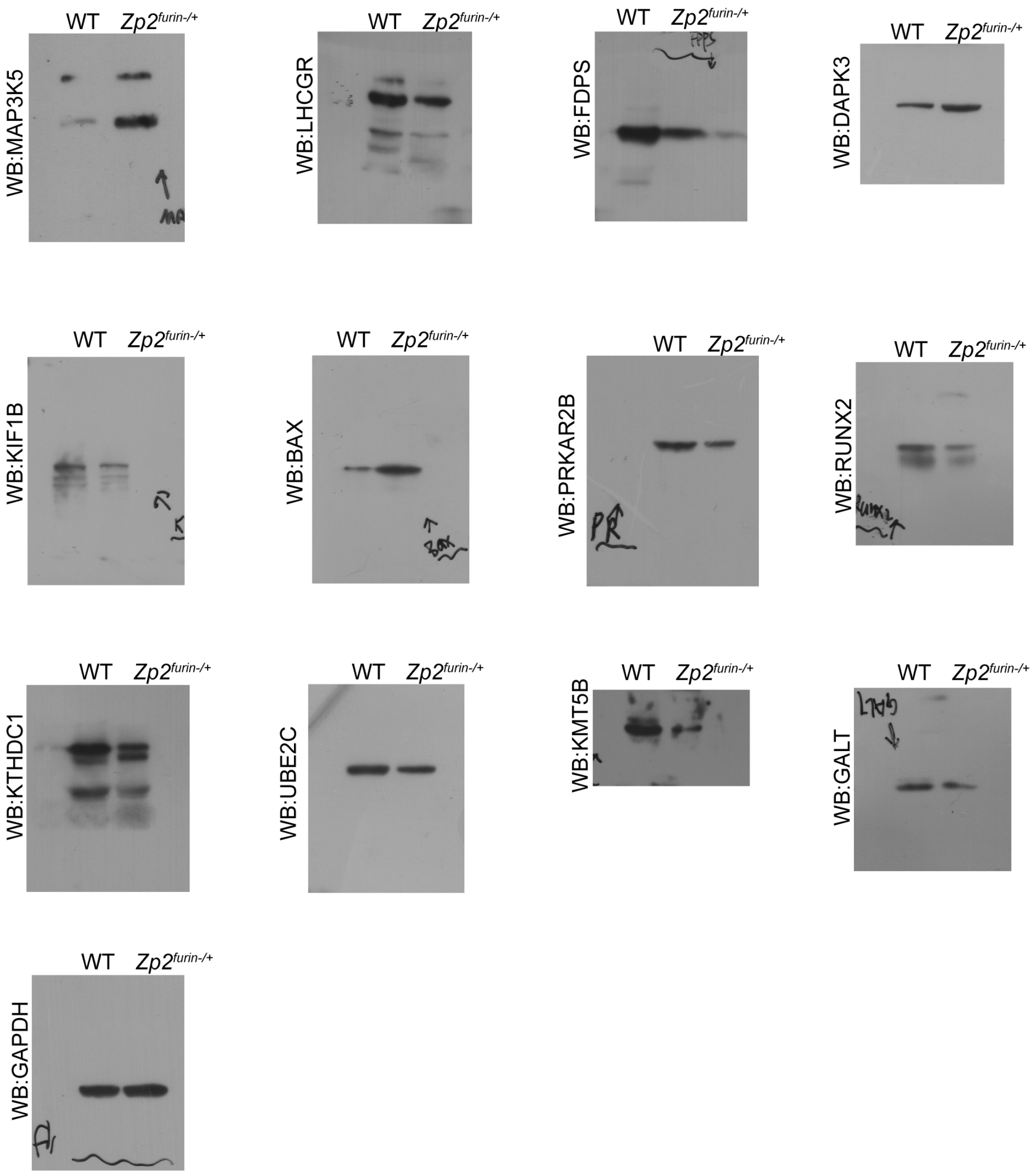
WT
Zp2furin-/+
WB:MAP3K5
WB:LHCGR
WB:FDPS
WB:DAPK3
WB:KIF1B
WB:BAX
WB:PRKAR2B
WB:RUNX2
WB:KTHDC1
WB:UBE2C
WB:KMT5B
WB:GALT
WB:GAPDH

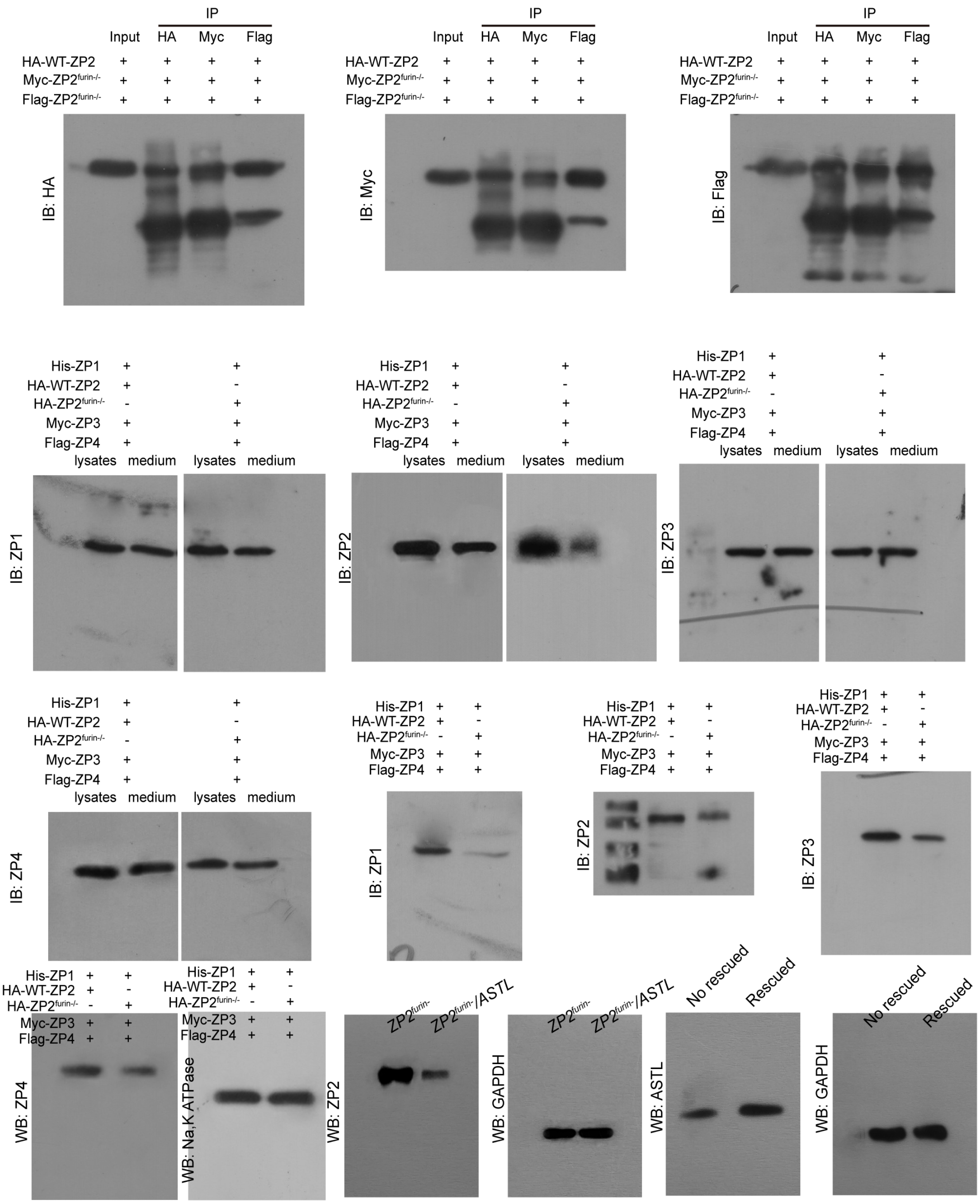
IP
Input
HA
Myc
Flag
HA-WT-ZP2
Myc-ZP2$^{furin-/-}$
Flag-ZP2$^{furin-/-}$
IB: HA
IB: Myc
IB: Flag
His-ZP1
HA-WT-ZP2
HA-ZP2$^{furin-/-}$
Myc-ZP3
Flag-ZP4
lysates
medium
IB: ZP1
IB: ZP2
IB: ZP3
IB: ZP4
WB: ZP4
WB: Na,K ATPase
ZP2^{furin-}
ZP2^{furin-}/ASTL
WB: ZP2
WB: GAPDH
No rescued
Rescued
WB: ASTL
WB: GAPDH
